# Challenges of combining field outcrops and E-logs to improve oil recovery in Cenomanian reservoirs, North Silah deep field, Western Desert, Egypt

**DOI:** 10.1038/s41598-026-46931-x

**Published:** 2026-08-19

**Authors:** Diana M. Helmy, Salah A. Omar, Fathy H. Mohamed, Adly H. D. El-Nikhely

**Affiliations:** 1 Petroleum Geology Master Program, Faculty of Science, Alex. University, Alexandria, 21511 Egypt; 2 Datalog Oilfield Service Company, Cairo, Egypt; 3 Geology Department, Faculty of Science, Alex University, Alexandria, 21511 Egypt; 4 Physics Department, Faculty of Science, Alex University, Alexandria, 21511 Egypt

**Keywords:** Outcrop facies log, Shoreface, Static model, Depositional model, Environmental sciences, Solid Earth sciences

## Abstract

The Upper Cretaceous Abu Roash Formation in Egypt’s Western Desert exhibits highly variable depositional environments, including fluvial, aeolian, tidal, and shoreface systems. This heterogeneity poses major challenges for predicting facies distribution, targeting new wells, and managing production. Conventional E-log–based interpretations typically focus on lithology, which lacks the spatial and geometrical resolution necessary for robust reservoir modeling. This study presents a novel workflow for 3D static modeling that integrates surface facies analogs with subsurface E-log data. Sedimentary structures and facies associations from the Abu Roash “G” Member type locality were analyzed to establish characteristic gamma ray (GR) responses for each facies. These GR signatures form a predictive interpretation scheme, enabling consistent facies identification in North Silah Deep Field wells. The interpreted facies logs were distributed using a deterministic modeling approach, constrained by the field’s conceptual depositional model and available reservoir data. The resulting static model incorporates porosity and water saturation, providing a detailed framework for volumetric calculations and supporting dynamic simulation. This methodology improves facies prediction in complex, heterogeneous reservoirs and offers a transferable approach for other stratigraphically and texturally diverse systems. By directly linking surface analogs to subsurface logs, it bridges the gap between sedimentological analysis and reservoir characterization, enhancing the accuracy of geological models and supporting more informed field development strategies.

## Introduction

Facies modeling is a critical component of reservoir characterization and the development of accurate static models. In the absence of seismic imaging or extensive core data, facies interpretation typically depends on available tools such as core data, Formation Micro-Imager (FMI) logs, and E-logs. Core data is essential for identifying depositional environments and facies associations, but it generally covers limited intervals and does not extend throughout the.

reservoir. FMI logs are highly useful for detailed facies and fracture analysis; however, they are not available in all fields or may only exist for limited intervals. E-logs, by contrast, are available for nearly all wells and cover entire reservoir intervals, making them a vital source of data. In conventional workflows, wireline logs are typically used to identify lithologies. However, because lithological units do not have consistent geometries, extrapolating lithology-based facies leads to random and unreliable results. This is why most 3D modeling software uses stochastic approaches, which rely on random distribution, rather than deterministic models based on geologic reality. In the absence of core and FMI data, characterizing Abu Roash “G” Member becomes particularly challenging. This raises several key questions:

1. What are the differences in depositional environments between the upper, middle, and lower members of the Abu Roash Formation?
2. What is the facies architecture for each environment in the field?
3. How can facies be captured from wireline logs?
4. How can sedimentological facies correlations replace lithofacies correlations?
5. What is the best technique for 3D well correlation versus 2D correlation?
6. How can a conceptual model be constructed based on field analogs and available data?
7. What is the optimal method for building a deterministic 3D model instead of a stochastic one?

Numerous reservoir studies have addressed the interpretation of facies from E-logs, either directly or by characterizing surface outcrops to understand reservoir architecture and rock properties across different depositional environments (e.g., Emery and Myers^1^^[,2^. Emery and Myers^1^, introduced a method for categorizing facies based on gamma-ray log shapes, dividing them into five groups. Terms such as “bell” or “funnel” shapes have since become standard in facies detection from E-logs. Phujareanchaiwon et al.^3^, applied this approach to capture lithology as interbedded sandstone and calcareous mudstone; however, they noted that lithofacies alone are insufficient to predict depositional geometry, as specific shapes are not defined for sandstone or other lithologies. Akbarzadeh et al.^4^, assumed a “box” shape for gamma-ray responses^5^, although this shape can encompass a wide range of facies from continental to deep-marine environments. Díaz-Curiel et al.^6^, used gamma-ray logs to estimate clay content while drilling and to account for the influence of wellbore conditions, such as the drill pipe and gravel pack, on the measurement. These limitations underscore the importance of detailed sedimentological facies analysis once the general depositional environments are well constrained.

The aim of this study is to use facies logs derived from this approach to construct a 3D static reservoir model and to populate key petrophysical parameters, such as porosity and water saturation, in order to support volumetric calculations and the targeting of new wells.

## Geological setting

### Sub-surface reservoir (North Silah deep)

**Fig. 1:**
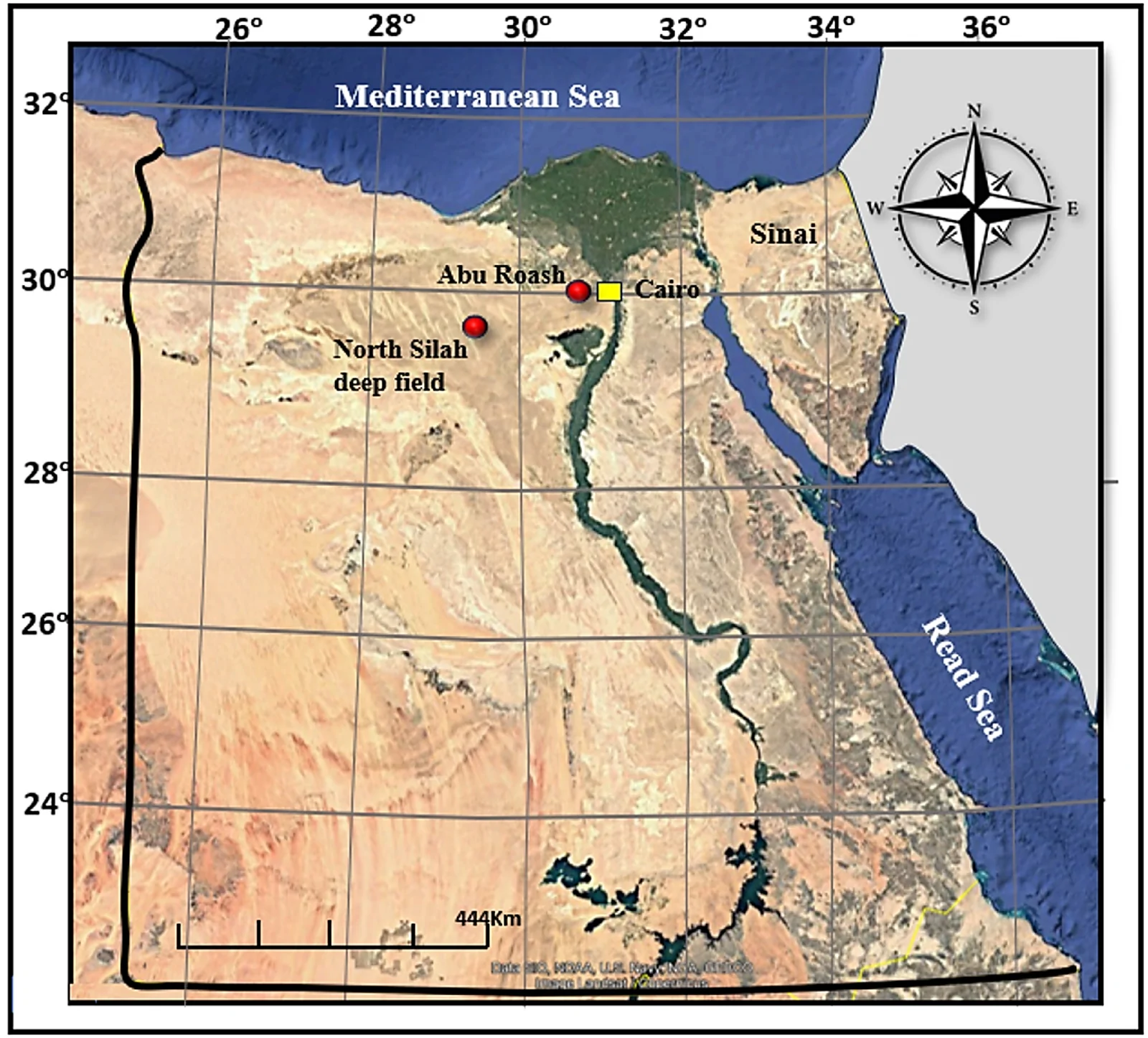
Location map of the study area in the Western Desert, Egypt, showing the Abu Roash and North Silah deep field locations. The base map is derived from Google Earth imagery (Imagery © Google, Data SIO, NOAA, U.S. Navy, NGA, GEBCO) and modified by the authors.

The sedimentary basins of Egypt’s Western Desert (Fig. 1) are located in the northern part of the country and form part of the Tethyan passive margin that developed following Jurassic–Early Cretaceous rifting^7,8^. The structural evolution of these basins involved several major tectonic phases: Jurassic Rift Phase: Initiated by NW–SE extension, forming early rift basins^9,10^. Continued Jurassic Rifting: NE–SW extension resulted from westward-propagating WNW–ESE rifting, producing both E–W and NE–SW trending basins such as the Abu Gharadig and Gindi Basins^11^. Early Cretaceous Extension: Continued NE–SW extension driven by the clockwise rotation of the African Plate relative to Eurasia caused E–W trending basin extension and the subsidence of basins like Abu Gharadig, accompanied by the formation of new NW–SE trending basins^12^. Late Cretaceous to Late Eocene Compression (~ 84–27 Ma): This phase was marked by major plate reorganization and the continued convergence of Africa and Eurasia, leading to tectonic inversion, folding, and regional uplift. The Abu Gharadig Basin—approximately 50 to 75 km wide and 330 km long—is among the most prominent Mesozoic extensional rift basins in northern Egypt^13^(Fig. 1). The Abu Roash Formation is unconformably overlain by the Campanian–Maastrichtian Khoman Chalk^14^(Fig. 3). The term “Khoman.

Chalk"was introduced by^15^ to describe the chalky limestone found west of Ain Khoman, south of the Bahariya Oasis. It is also recorded near the village of Abu Roash and around the El-Hassana Dome. The Khoman Chalk consists mainly of white chalky limestone with chert bands in the upper part. In some locations, it becomes dolomitized along joints and fault traces^16^. There is no clear evidence of Paleocene–Lower Eocene rocks in the Abu Roash area, with the exception of data collected by^17^, who dated samples from the Gebel Jiran El-Ful scarp based on planktonic foraminifera.

Exposed Oligocene clastic and associated basaltic sheets along the flanks of the Abu Roash structures are linked to rift-related volcanism and correlate with the Gebel Qatrani Formation^18,19^. Quaternary rocks in the area consist mainly of terrestrial classics that blanket the topographic lows.

Data from older stratigraphic units were obtained from Abu Roash-1 and Abu Roash-2 wells. The Abu Roash Formation unconformably rests on the Lower Cenomanian Bahariya Formation, which in turn overlies the Lower Cretaceous Alam El-Bueib, Alamein, and Kharita formations. Deeper units include Upper Paleozoic (Carboniferous–Permian?) deposits, which rest unconformably above the Precambrian Basement, and are themselves overlain by Jurassic formations such as Ras Qattara, Wadi El Natrun, Khatatba, and Masajid(Fig. 3)^19^.

**Fig. 2:**
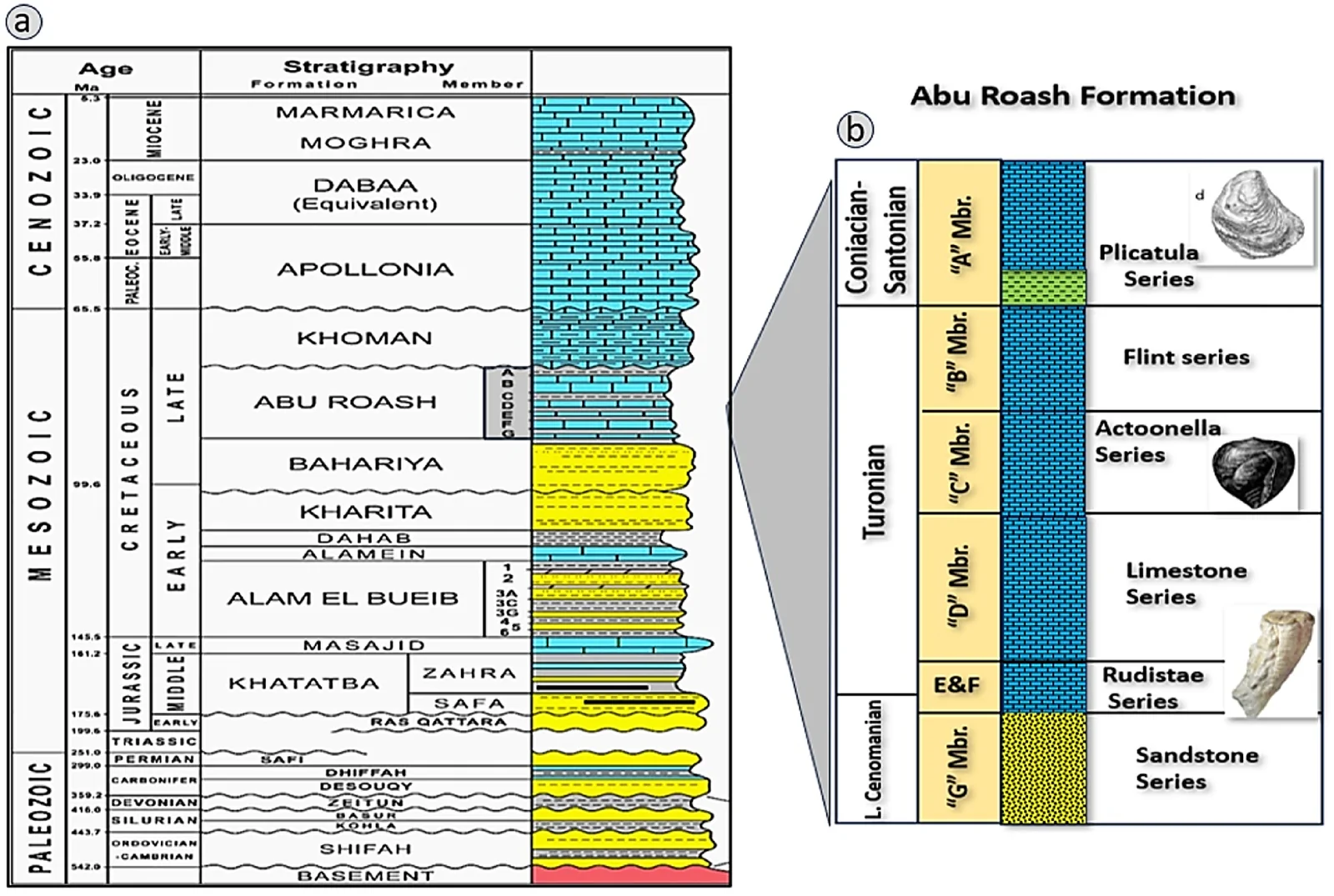
(**a**) Generalized geological column of the Western Desert, Egypt (after Ebraheem et al.^20^). (**b**) Stratigraphic framework showing the detailed surface and subsurface characteristics of the Abu Roash Formation, including its lithostratigraphic subdivisions.

### Surface analogy (Abu Roash area)

**Fig. 3:**
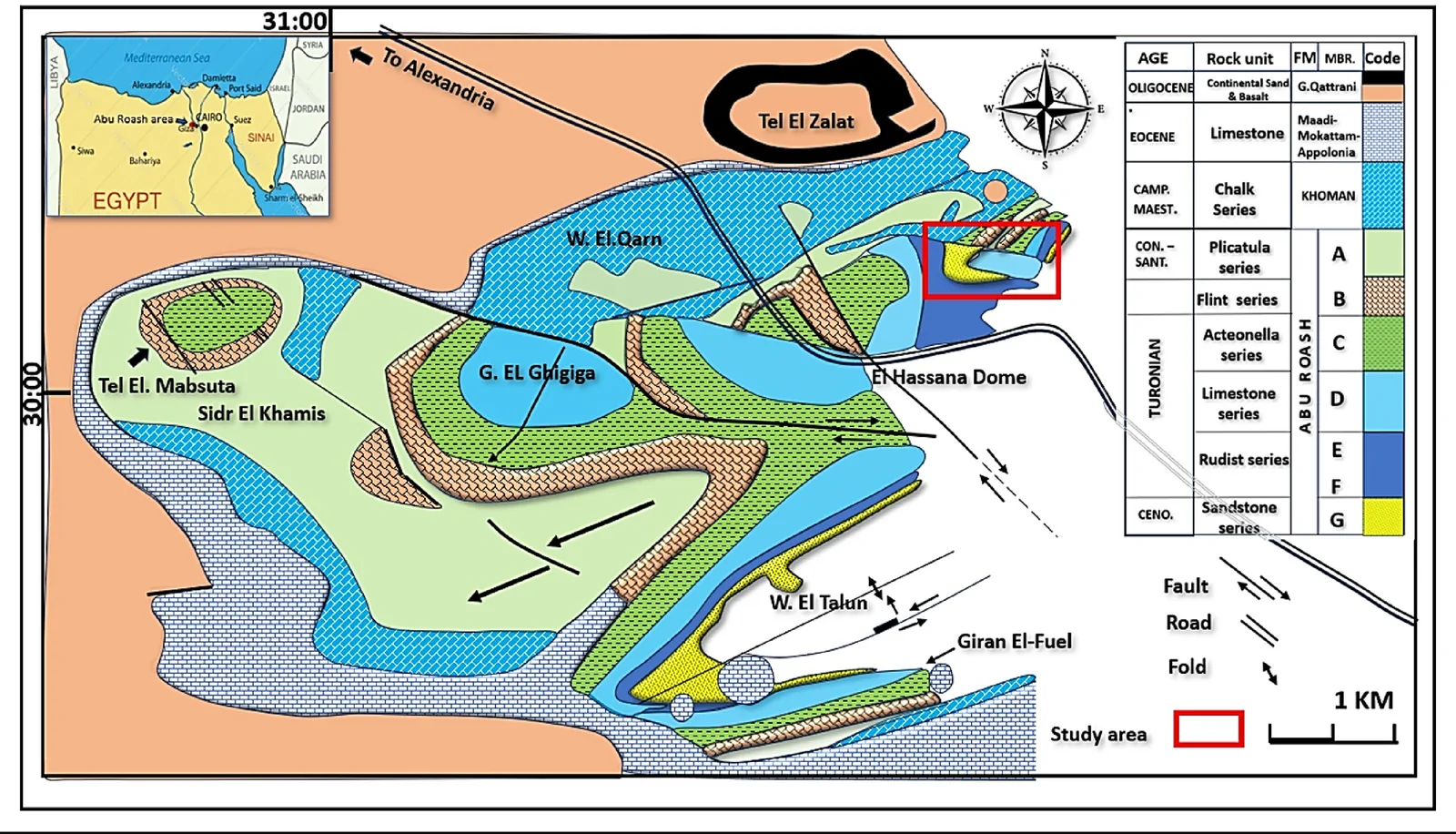
Geological map of the Abu Roash area illustrating regional lithological units and the location of the study area highlighted by the red box (modified after Abu Khadra et al., 2005).

The Abu Roash area is located north of the Giza Pyramids at coordinates 30° 2′ 44.59″ N and 31°14′ 24.71″ E (Fig. 3). The Abu Roash Formation is considered to be of Upper Cenomanian to Turonian (Senonian) age and is subdivided into seven members, named from top to bottom: A, B, C, D, E, F, and G. The Abu Roash “G” Member is composed of interbedded limestone and shale, with sandstone layers. It also contains a dolomite unit, which is thought to have been deposited in a sabkha or nearshore environment. Core analysis of the Abu Roash “G” Member revealed fair porosity and permeability. Lithology is primarily carbonate, consisting of dolomite and limestone with minor sandstone intercalations^21^. The Abu Roash “G” Member was deposited in a nearshore environment. In contrast, Saleh et al.^21^, provided a more detailed interpretation, suggesting that the upper and middle sections were deposited in a deltaic environment, whereas the lower section reflects tidal depositional conditions.

**Fig. 4:**
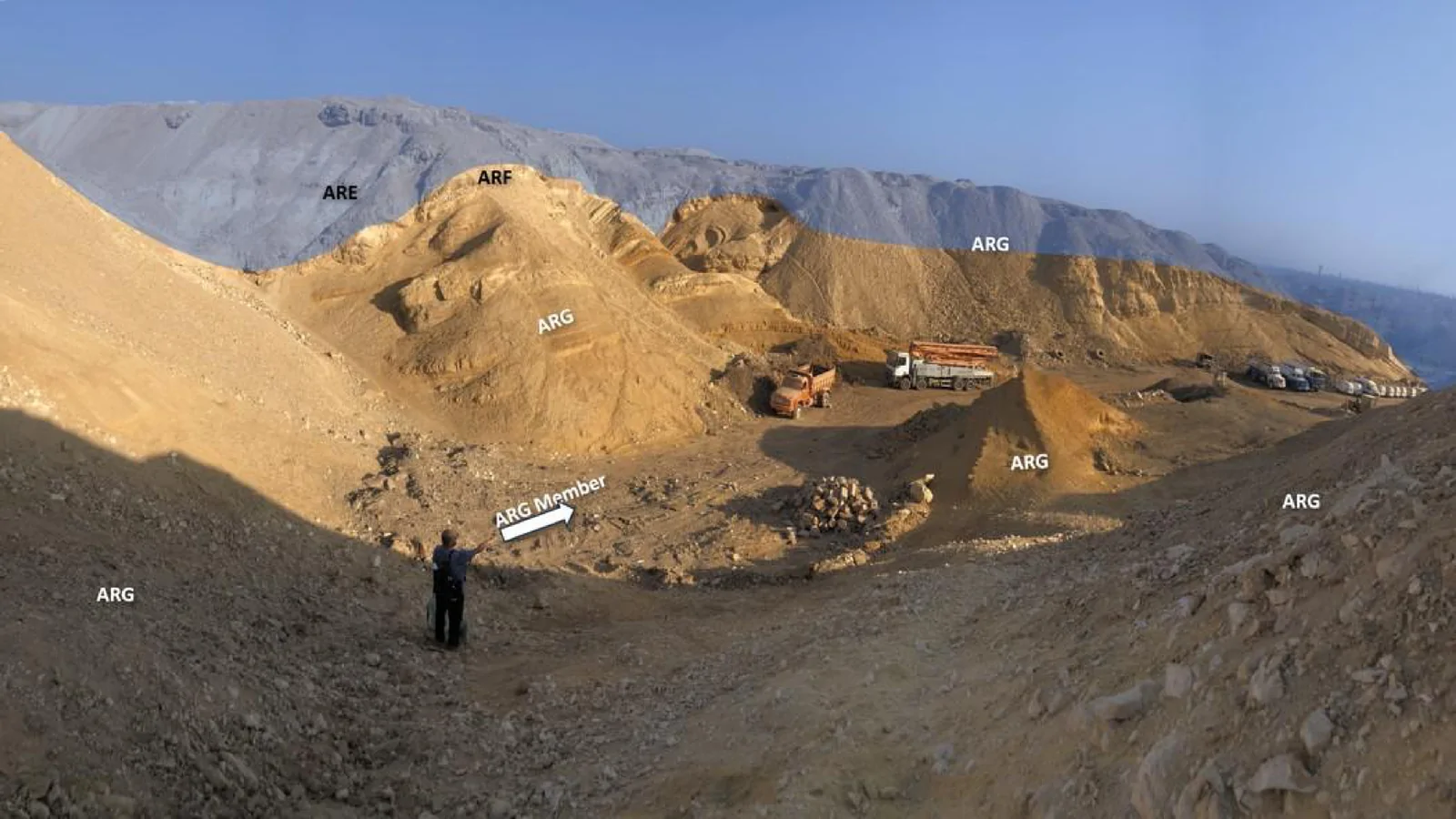
Relationship between the Abu Roash “G” Member and the “E” & “F” Members in the study area. The Abu Roash “G” is characterized by the presence of yellow sandstone interbedded with dolomitic limestone. In contrast, the overlying “E” and “F” Members are dominated by carbonate lithofacies, forming predominantly carbonate reservoirs.

The Abu Roash “G” Member consists mainly of thin intercalations of yellow to brown sandstone and mudstone (Fig. 4). In contrast, the overlying “E” & “F” Members are dominated by carbonate lithologies. Abu Roash “F” Member, in particular, contains abundant Rudists. The succession indicates a shallow-marine carbonate platform with fair porosity and permeability, where the lithology is dominated by carbonate rocks (dolomite and limestone) with minor sandstone intercalations. The Upper and Middle parts of the member are interpreted as deltaic deposits, whereas the Lower part is suggested to represent a tidal-complex environment. The Abu Roash “G” Member is mainly a thin intercalation between yellow to brown sandstone and mudstone. Meanwhile, the other Members (“E” & “F”) are mainly carbonate with abundant of rudists in Abu Roash “F” overlaying Abu Roash “G” Member in the entrance of Wadi Abu Roash Area.

## Materials and methods

**Fig. 5:**
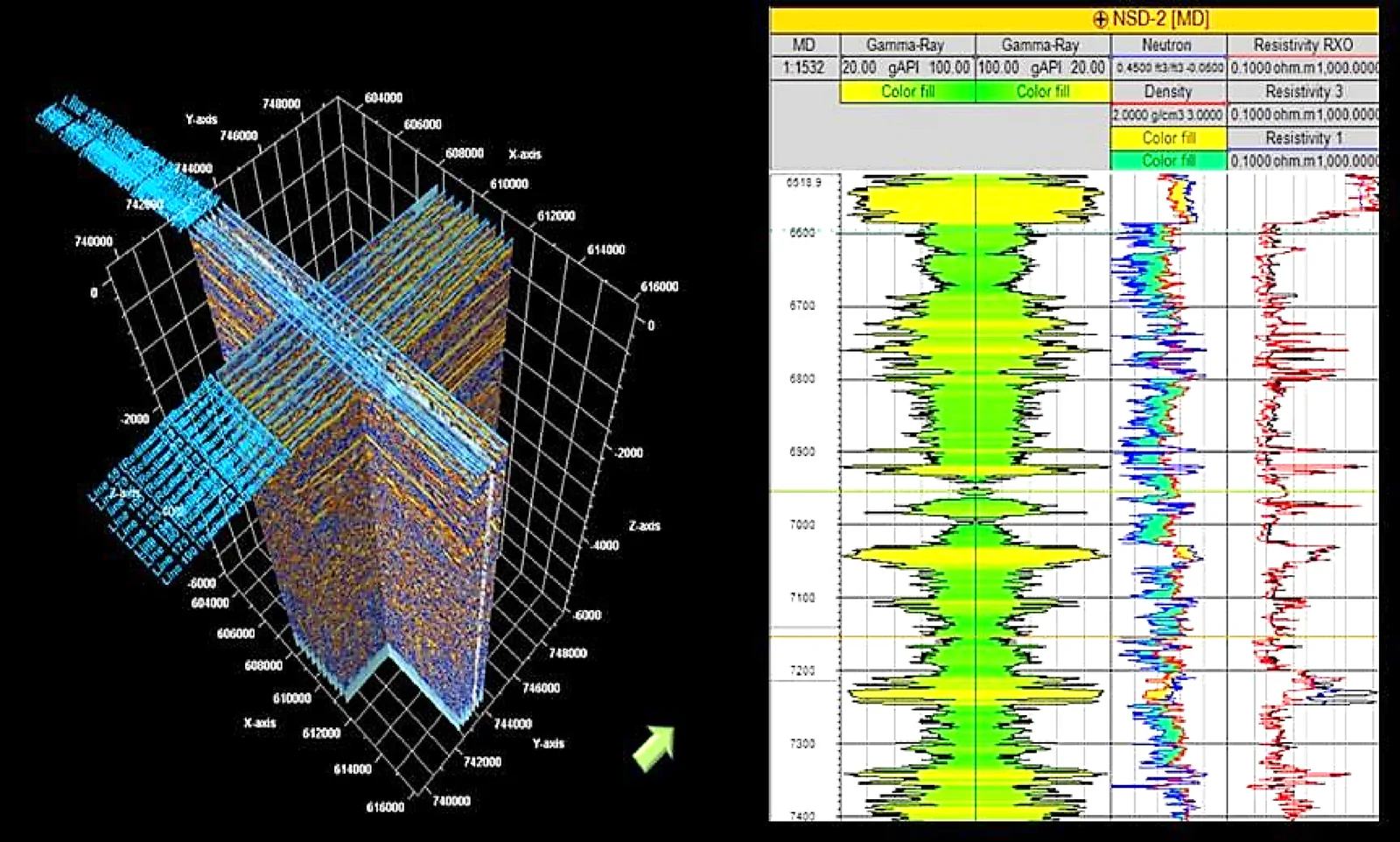
The available dataset includes 20 2D seismic lines and petrophysical logs for four wells (e.g., NSD-2). The dataset was interpreted and visualized by the authors using Petrel.

The present study aims to develop a deterministic facies model for the Abu Roash “G” Member in the North Silah Deep Field. Due to the limited availability of core data, FMI logs, and sedimentological studies, applying a field-analogy approach can help reduce these uncertainties. The available dataset (Fig. 5) includes: Twenty 2D seismic lines and petrophysical raw data (Gamma Ray, Neutron, Density, and Resistivity logs) from four wells: NSD-2 well, NSD-2-2 well, NSD-1–7 well, NSD-1X well.

Traditional E-log analysis involves constructing lithological logs for use in 3D facies modeling. However, this approach faces a fundamental challenge: there is no distinct geometry for lithological units such as sandstone, siltstone, or limestone. In contrast, sedimentological facies such as channels, deltas, or shoreface deposits have recognizable depositional patterns and geometries, making them more suitable for realistic static modeling. Myers and Bristow^22^. introduced the concept of identifying facies directly from E-logs. Their methodology uses five characteristic gamma ray log shapes; each associated with specific sedimentological environments. While this technique marked a breakthrough, one limitation remains: each gamma ray shape may correspond to multiple depositional environments, ranging from continental to deep marine settings. This paper proposes a new methodology for capturing sedimentological facies from field outcrops and matching them with subsurface E-log data to guide conceptual modeling. The method was applied to Upper Cretaceous outcrops in two different locations in Egypt.

The approach can be summarized as follows:

1. Field section selection: Identify the most complete and representative outcrop section.
2. Facies documentation: Record lithology, sedimentary structures, trace fossils, and facies associations for each interval.
3. Sample analysis: Collect core and thin section samples from intervals of interest for laboratory analysis.
4. Gamma ray prediction: Simulate expected gamma ray shapes for each facies based on their depositional mechanisms. For example:
  Channel deposits: Scour underlying beds, resulting in sharp contacts on E-logs and typically show a fining- or coarsening-upward trend.
  Braided systems: Exhibit sharp basal contacts and uniform grain sizes, producing cylindrical gamma ray profiles.

5. Field spectrometry: hand-held gamma-ray called gamma-ray spectrometry G512) were used for recording natural radionuclide radiations, recording energy windows centered on the 1461 keV (^40^K), 1765 keV (^214^Bi) and 2615 keV (^208^Tl) photo-peaks for the estimation of K, U, and Th concentrations, respectively.
6. Different measurement points were selected for each facies type and combined with the simulated gamma-ray curve for the corresponding facies, resulting in proposed gamma-ray logs for each facies type. This technique, supported by previous studies^22^, facilitates the correlation of outcrop-derived facies with subsurface gamma-ray responses.
7. Data normalization: Integrate and normalize spectrometer readings to produce representative gamma ray logs for each facies.
8. E-log calibration: Use these field-derived gamma ray profiles to interpret facies from E-log data in the study wells.
9. Facies log development: Construct new facies logs for the study wells using this calibrated gamma ray scheme.
10. Subsurface matching: Align field-based facies logs with those from NSD-2 and extend interpretation to NSD-2-2, NSD-1X, and NSD-1–7 wells.
11. 3D distribution: Apply the interpreted sedimentological facies for 3D reservoir modeling.
12. Petrophysical integration: Distribute porosity and water saturation properties within the model, guided by the new facies’ interpretation.
13. Well targeting: Use the final static model for identifying prospective zones for future drilling.

## Results

**Fig. 6:**
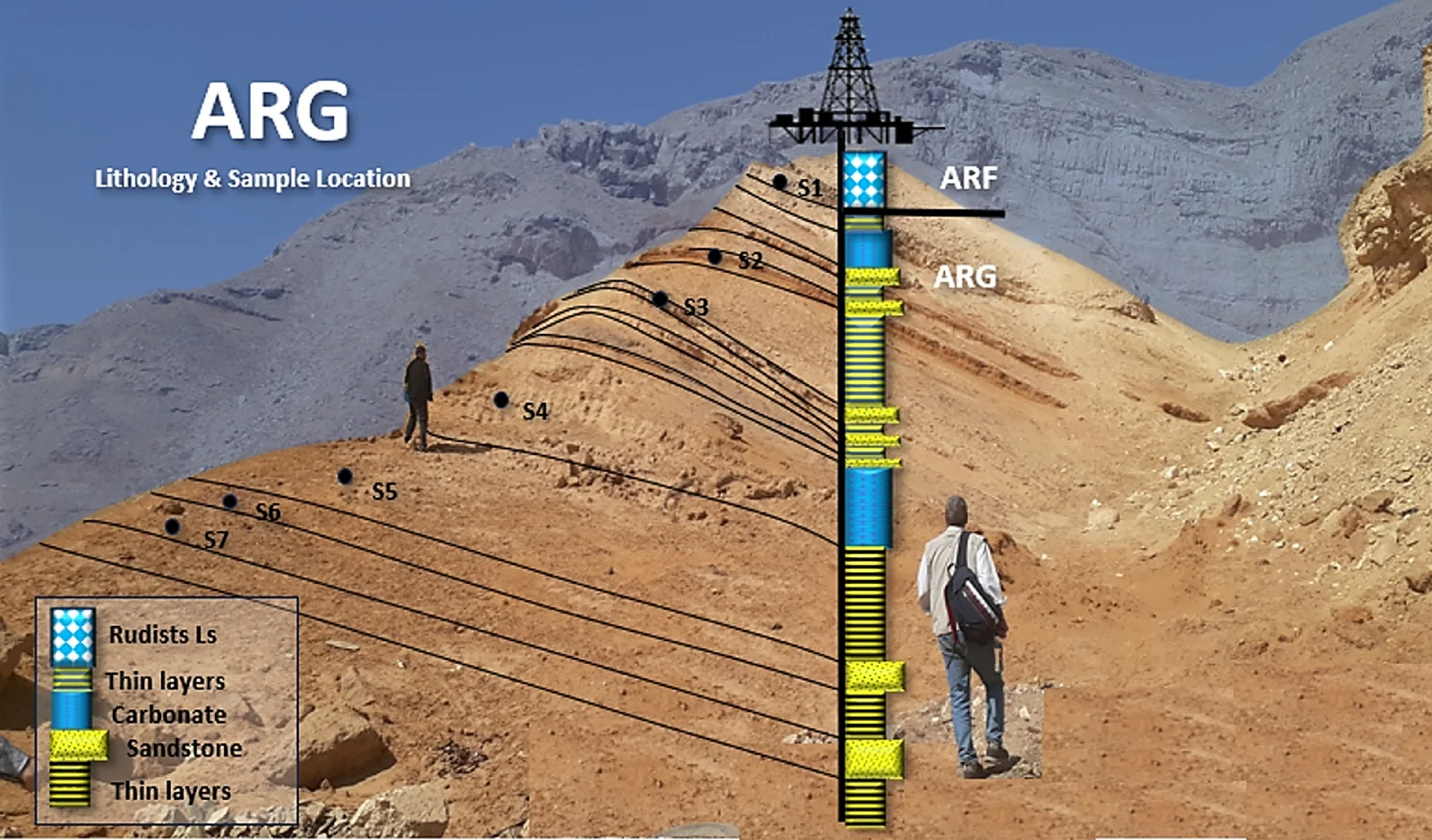
Lithology log for the max thickness of Abu Roash “G” Member in the Abu Roash Area showing different lithology types (Massive Sandstone, Massive Carbonate and Thin Layers). The Letter “**S**” represents the location of collected sample for lab analysis.

**Fig. 7:**
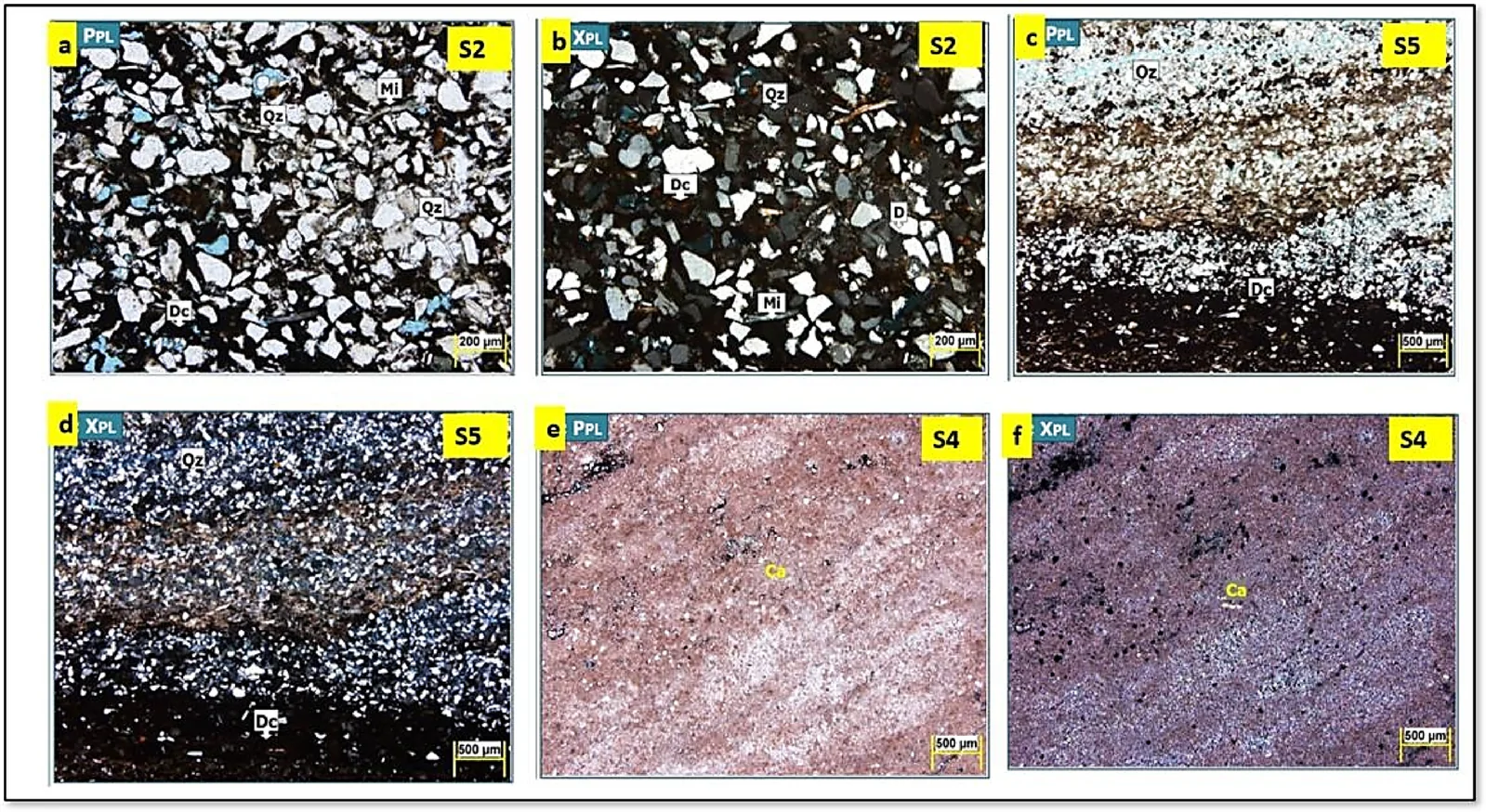
Photomicrographs of the studied siliciclastic and carbonate facies. (**a**) Plane-polarized light (PPL) photomicrograph of sample S2 showing a quartz arenite composed mainly of subangular quartz (Qz) grains with minor muscovite (Mi). The sandstone is fine- to medium-grained and moderately sorted, with limited clay matrix (Dc). (**b**) Cross-polarized light (XPL) image of the same facies (S2) showing quartz grains with undulose extinction and minor clay occupying intergranular pores. (**c**) PPL photomicrograph of sample S5 displaying a quartz wacke characterized by alternating quartz-rich (Qz) and clay-rich (Dc) laminae forming well-defined sedimentary lamination. (**d**) XPL image of S5 highlighting quartz-dominated laminae and clay-rich layers expressed as darker brownish bands. (**e**, **f**) PPL and XPL photomicrographs of sample S4 illustrating a carbonate facies classified as crystalline calcite, dominated by crystalline calcite cement.

Based on the methodology described above, the most suitable field location (Fig. 6) was selected based on the maximum observed thickness of the Abu Roash “G” Member. A pseudo well section was constructed and divided into multiple intervals according to lithological changes: massive sandstone, massive carbonate, and thin interbedded sand/shale layers. The sandstone interval is homogeneous in both grain size and thickness and is characterized by sharp contacts at both the top and base. Seven samples were collected from this section for laboratory analysis. This study integrates field-collected samples and sedimentary structures. Rock samples were investigated using petrographic analysis (Fig. 7), following the sandstone classification of Pettijohn et al.^23^, and the carbonate textural classification of Dunham^24^.

Four key carbonate markers were identified: 

(1) Top Marker: Located at the contact between the Abu Roash “G” and “F” members.

(2) Two middle markers of extensive carbonate beds that divide the Abu Roash “G” into three intervals (upper, middle, and lower). These are often referred to as Intra- Abu Roash “G” transgressive marker beds^25^. 

(3) Basal Marker: Marks the transition between the ARG and the underlying Bahariya Formation.

The maximum thickness section (Fig. 6) was documented using a conceptual lithological log, illustrating transitions between massive sandstone, massive carbonate, and thin layered interbedding.

Each sampling location is denoted by the letter “S” for reference in the lab analysis (Fig. 7). Based on field analogs, facies associations, and thin section analysis, five main sedimentary facies were defined within the Upper Cenomanian Abu Roash “G” Member: (FS1: Shoreface Sandstone, FS2: Sand Flat Facies, FS3: Mixed Flat Facies, FS4: Mud Flat Facies and FS5: Carbonate Facies).

### Shoreface & facies association (FS1)

#### Description

**Fig. 8:**
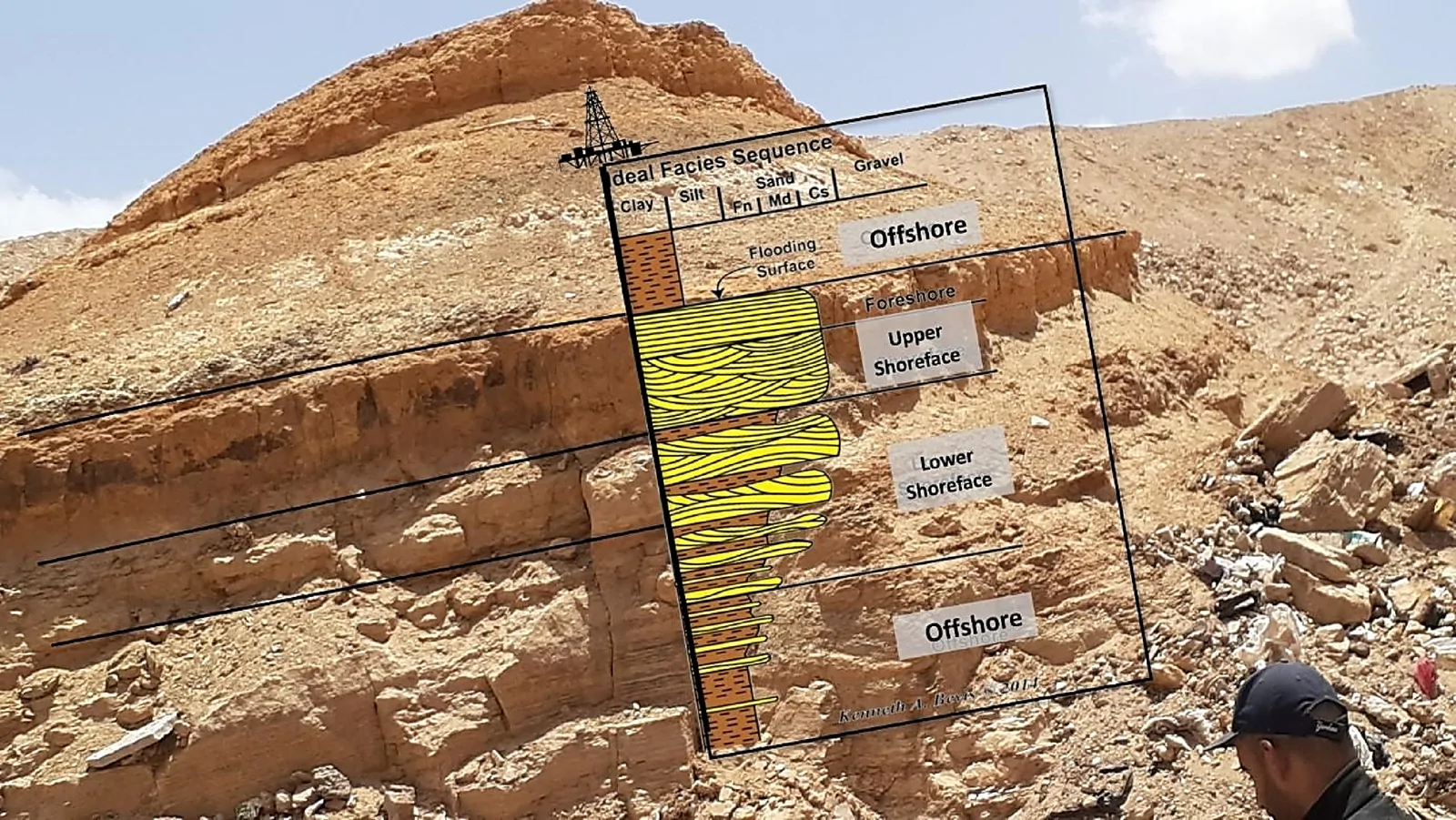
Field photograph illustrating shoreface types and corresponding grain-size profile from the study area, showing the vertical transition from offshore to lower, middle, and upper shoreface. The observed coarsening-upward trend in grain size is consistent with established field analogs and can be used to simulate the expected Gamma Ray log response, which typically decreases upward due to increasing sand content. (Diagram adapted from^26^.

The shoreface environment comprises marine sandstone deposits accumulated parallel to the shoreline and extending seaward to water depths of approximately 20 m (Fig. 8). Variations in grain size, sorting, sedimentary structures, and trace-fossil assemblages constitute the primary criteria for subdividing the shoreface succession. Accordingly, the shoreface is differentiated into upper, middle, and lower zones. The upper shoreface is dominated by coarse- to medium-grained, well-sorted, texturally mature sandstones that are typically homogeneous and laterally persistent. These deposits reflect high-energy wave and swash-zone processes operating proximal to the shoreline and are commonly characterized by low mud content and high net to-gross ratios. The middle shoreface is composed mainly of medium- to fine-grained, moderately sorted sandstones, with an increasing proportion of heterolithic bedding and mud drapes in the seaward direction. Sedimentary structures indicate a reduction in hydrodynamic energy relative to the upper shoreface. The lower shoreface is characterized by fine-grained, poorly to moderately sorted sandstones that grade downward into sandy mudstone and mudstone. These deposits reflect lower-energy storm-dominated conditions below the fair-weather wave base, with increased clay content, bioturbation intensity, and vertical grain-size fining.

#### Facies characteristics and log interpretation guidance

**Fig. 9:**
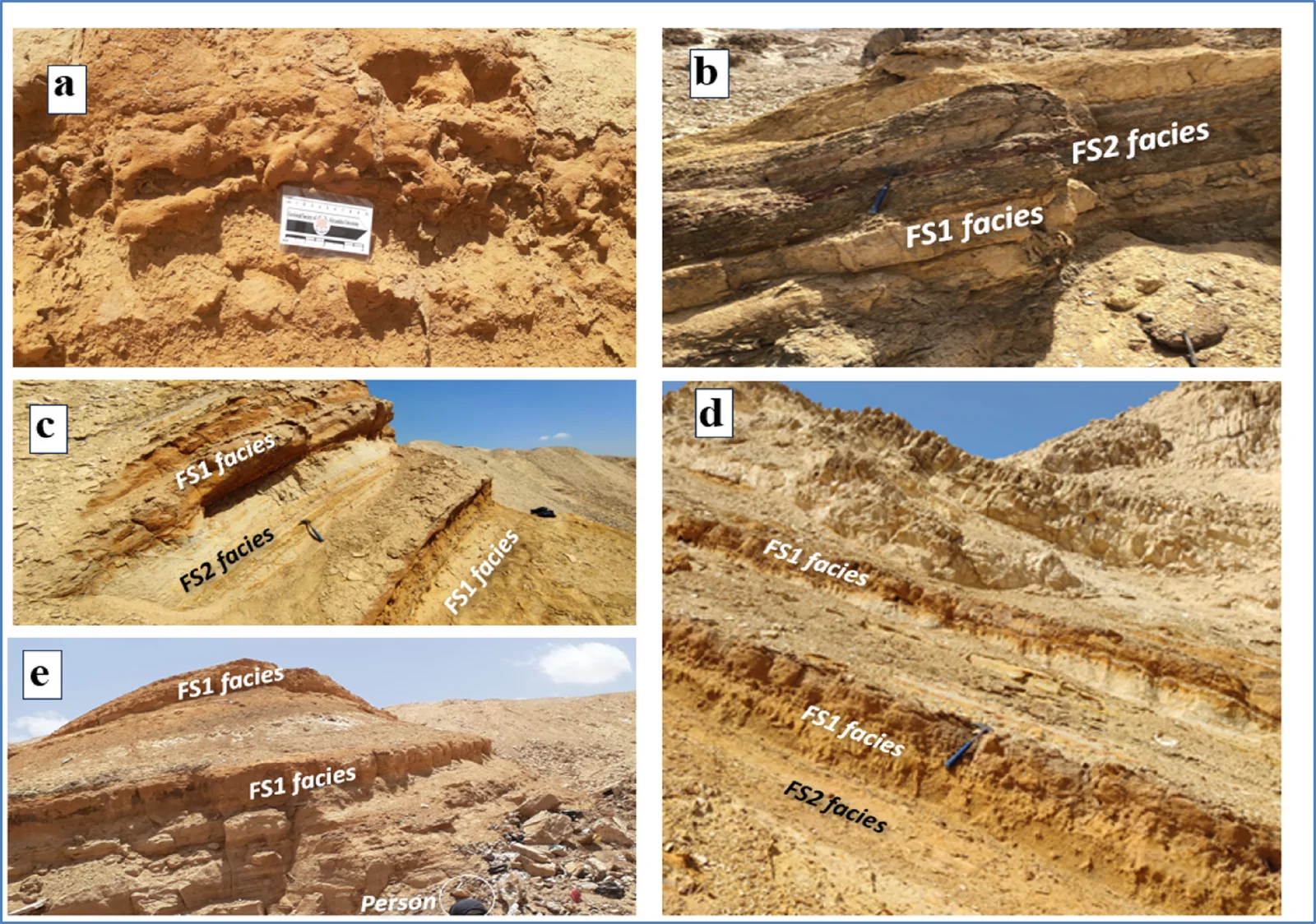
Field photograph of Shoreface (FS1) facies: (**a**) Thalassinoides ichnospecies marking the transition between upper and middle shoreface; (**b**–**e**) Thin shoreface layers are situated between tidal flat deposits.

**Fig. 10:**
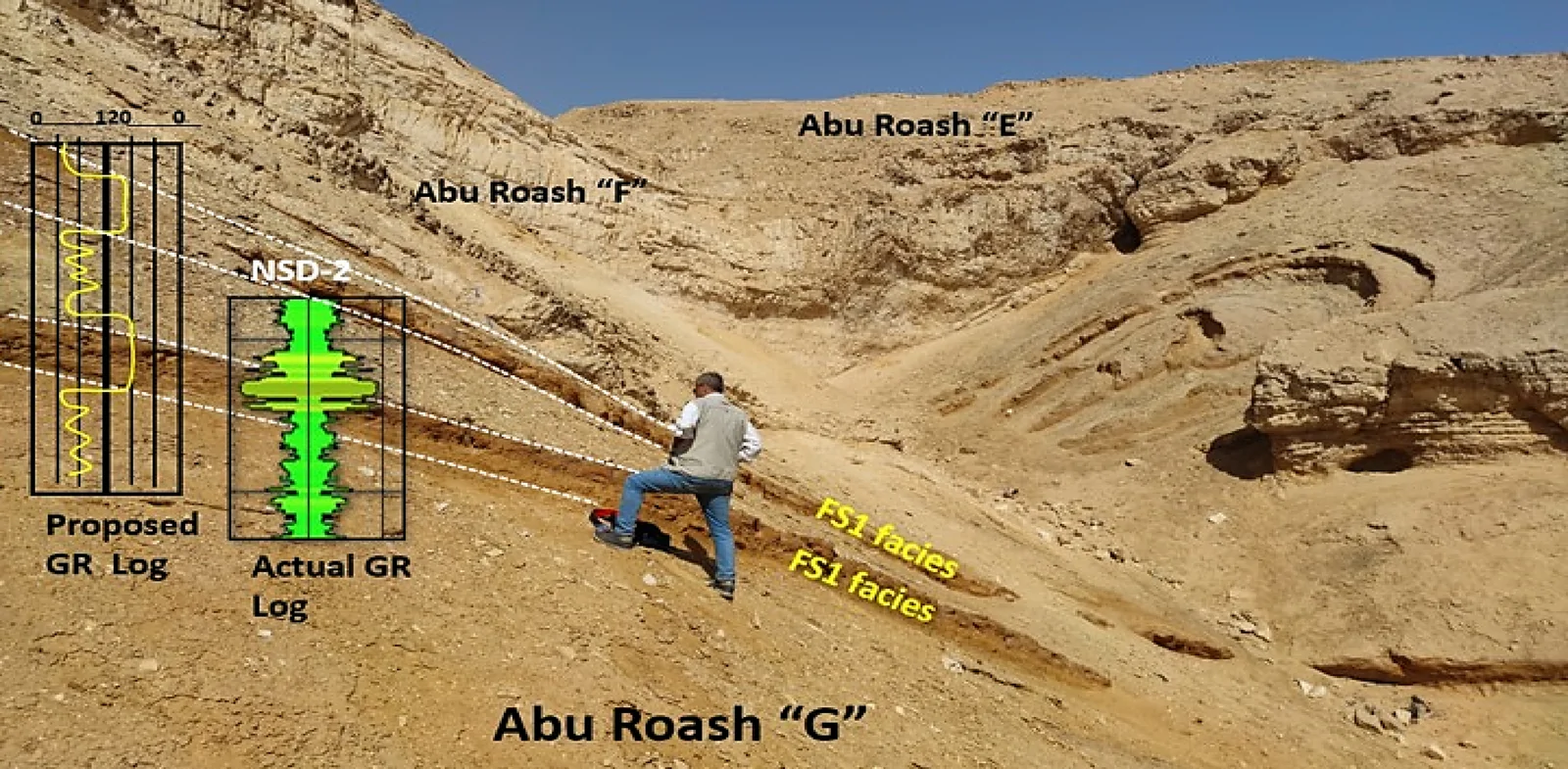
Predicted Gamma Ray log response for shoreface cycles within the Abu Roash “G” Member in the Abu Roash area. The NSD-2 well log displays a similar blocky Gamma Ray signature, supporting the shoreface interpretation.

Key features of the FS1 facies that can assist in facies log construction include: (1) Sharp contacts at both the top and base of the sandstone body. (2) Each shoreface type (upper, middle, lower) is homogeneous in grain size and thickness. (3) Wide lateral extent. (4) The upper shoreface may transition laterally into middle or lower shoreface facies. (6) The upper shoreface can sometimes exhibit dolomitic limestone or calcareous capping due to lagoonal influence. (7) Middle and lower shoreface zones characterized by hummocky cross-stratification as a result of storm activity. (8) Cross-bedding types vary depending on slope geometry—planar, horizontal, or trough types may occur. (9) The *Thalassinoides* ichnospecies is a diagnostic indicator of middle and lower shoreface deposits. (10) These characteristics are visually documented in thin sections and field profiles (Figs. 9 and 10).

#### Gamma ray log interpretation

Using the new field-based approach, the gamma ray (GR) response of the shoreface facies is typically blocky or box-shaped with low GR values, reflecting clean sandstone bounded by sharp contacts. It is not always necessary for upper, middle, and lower shoreface units to occur together in one section; the absence of one or more may reflect rapid tectonic changes or sea-level fluctuations. Comparison between the predicted gamma ray shape from the field and the actual GR log from the NSD-2 well (Fig. 10) shows strong correlation, particularly the blocky signature indicative of shoreface deposits.

### Tidal flat facies (FS2)

The tidal complex facies are among the most dominant facies observed in outcrops across the Abu Roash area. Even other facies types, such as shoreface and carbonate facies, show signs of tidal influence. Tidal environments can be recognized by specific sedimentary structures including bundled cross bedding, herringbone structures, flaser bedding, wavy bedding, and lenticular cross bedding.

#### Field observations: tidal flat structures

Tidal flat facies represent distinct sedimentary environments shaped by the alternating rise and fall of tidal waters. These coastal settings undergo regular wetting and drying, producing characteristic depositional patterns. The resulting sedimentary features record the interplay between tidal energy and sediment supply.

**Fig. 11:**
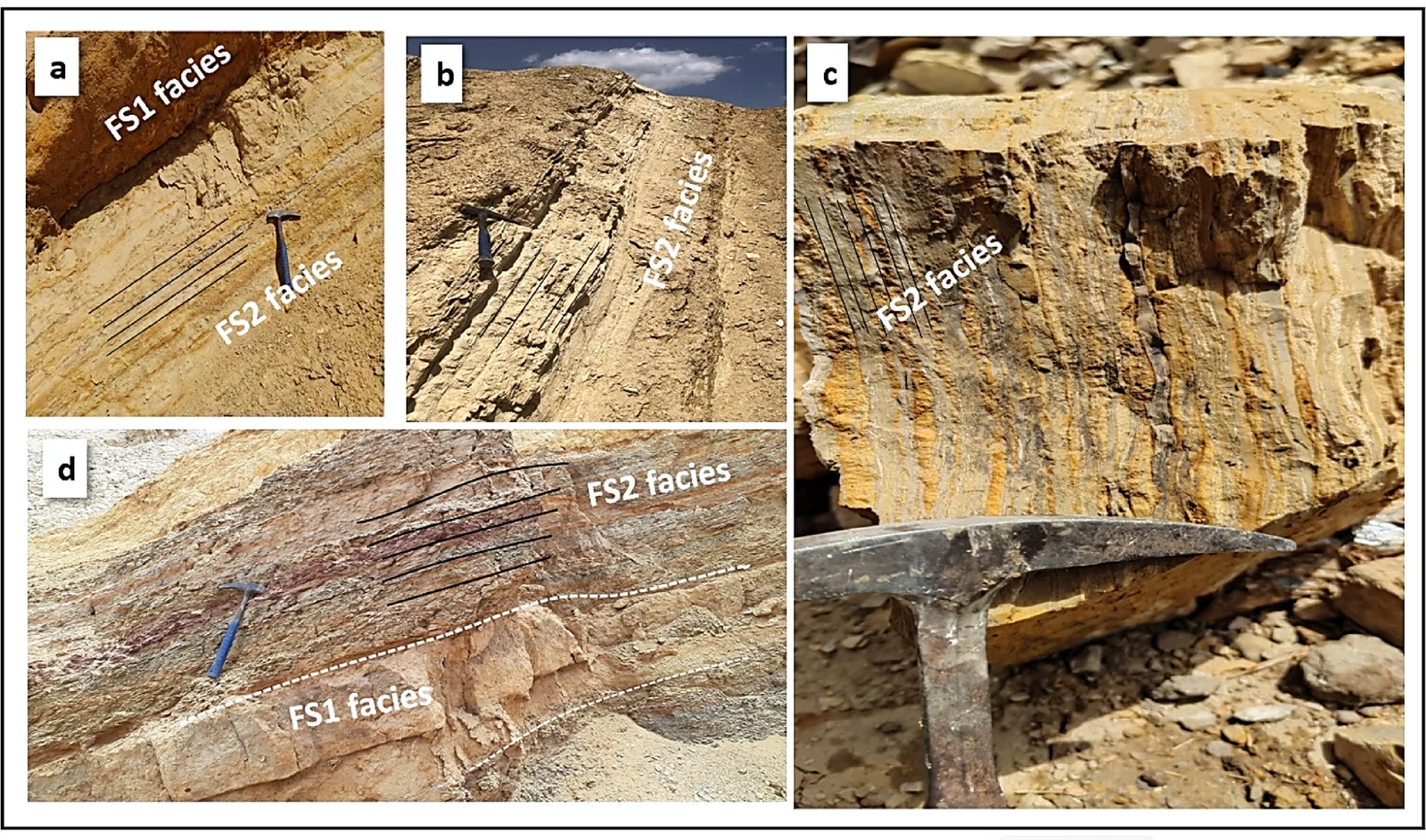
Field analogy for tidal facies in the Abu Roash “G” Member, displaying bundled cross bedding (black lines) as a key indicator of tidal influence. (**a**) Thin carbonate mudflat layer. (**b**) Thick sandflat succession showing well-developed cross bedding. (**c**) Different types of tidal bundles possibly formed during spring-tide conditions within sandstone beds. (**d**) Shoreface deposits interbedded with tidal-flat facies, reflecting transitional environments.

Tidal flat facies are typically divided into three main subtypes, each reflecting unique environmental conditions: (1) Mudflat Facies: dominated by fine-grained sediments (mud and silt). (2) Smooth, planar surfaces and commonly display desiccation cracks formed during exposure. (3) Sandflat Facies: composed of coarser sediments (typically sand), characterized by ripple marks and cross-bedding and formed under stronger tidal currents. (3) Mixedflat facies: interbedding of mud and sand represents transitional zones where tidal energy fluctuates. Each type of tidal flat facies preserves a distinct stratigraphic and sedimentological. signature. These signatures provide valuable information for interpreting ancient coastal systems and reconstructing paleoenvironmental conditions (Fig. 11).

#### Gamma ray log interpretation

**Fig. 12:**
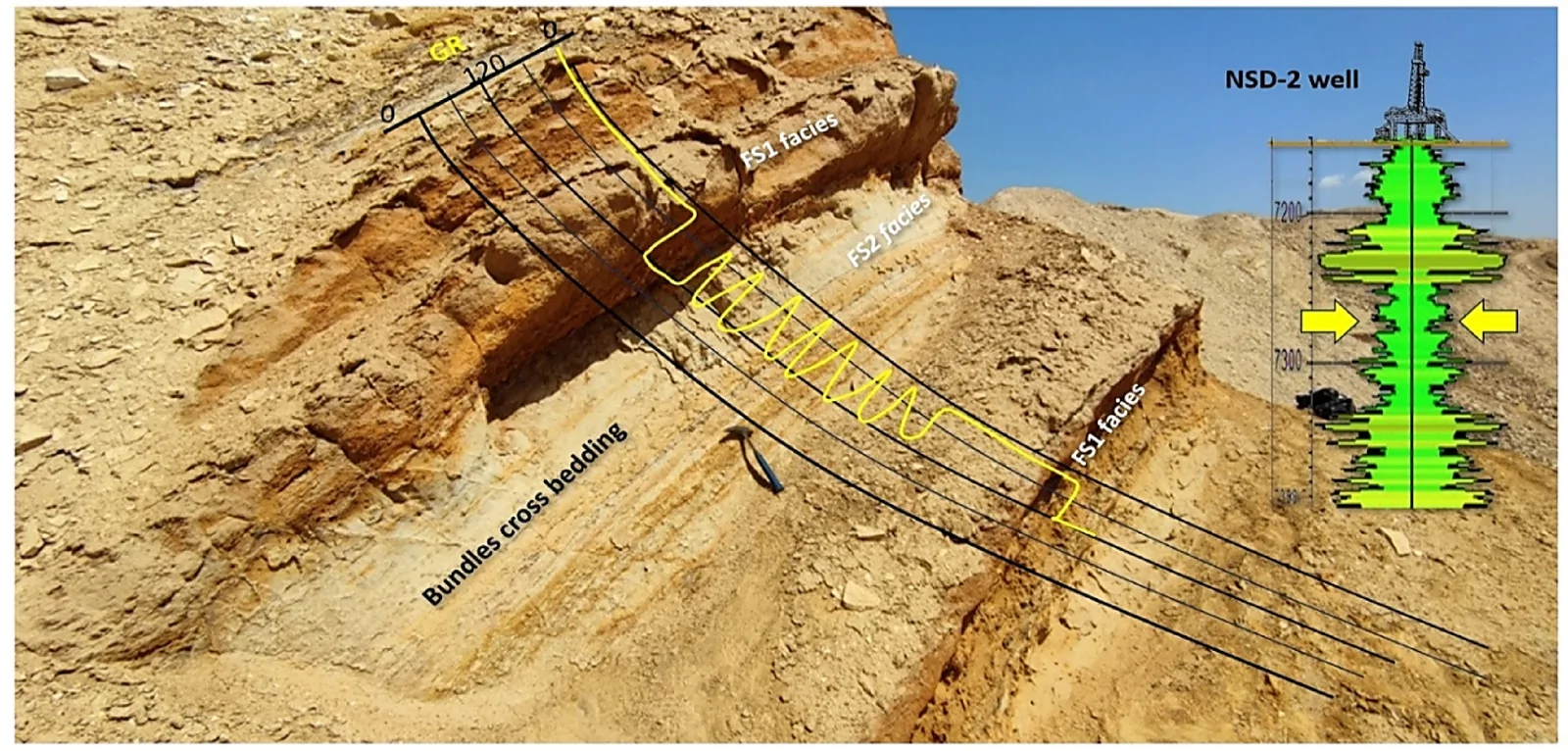
Field photograph illustrates the predicted Gamma Ray log for tidal flat environments, represented by the FS2 facies. Inset is the actual Gamma Ray log for NSD-2 well for comparison

In tidal flat settings, gamma ray log responses (Fig.12) are often variable, reflecting the mixed lithologies and repetitive bedding patterns. For instance, sand-rich intervals (sandflats) generally show lower GR values, while muddier intervals (mudflats) produce higher GR readings. The bundled cross bedding, a key sedimentary structure in tidal environments, can create repetitive GR fluctuations that are useful for facies recognition. This facies type can often be misinterpreted in lithological logs, but the new sedimentological approach allows for a more accurate and consistent facies interpretation using E-logs. As demonstrated in the NSD-2 well, gamma ray log profiles aligned well with field predictions.

### Carbonate facies (lagoonal lime-mud)

**Fig. 13:**
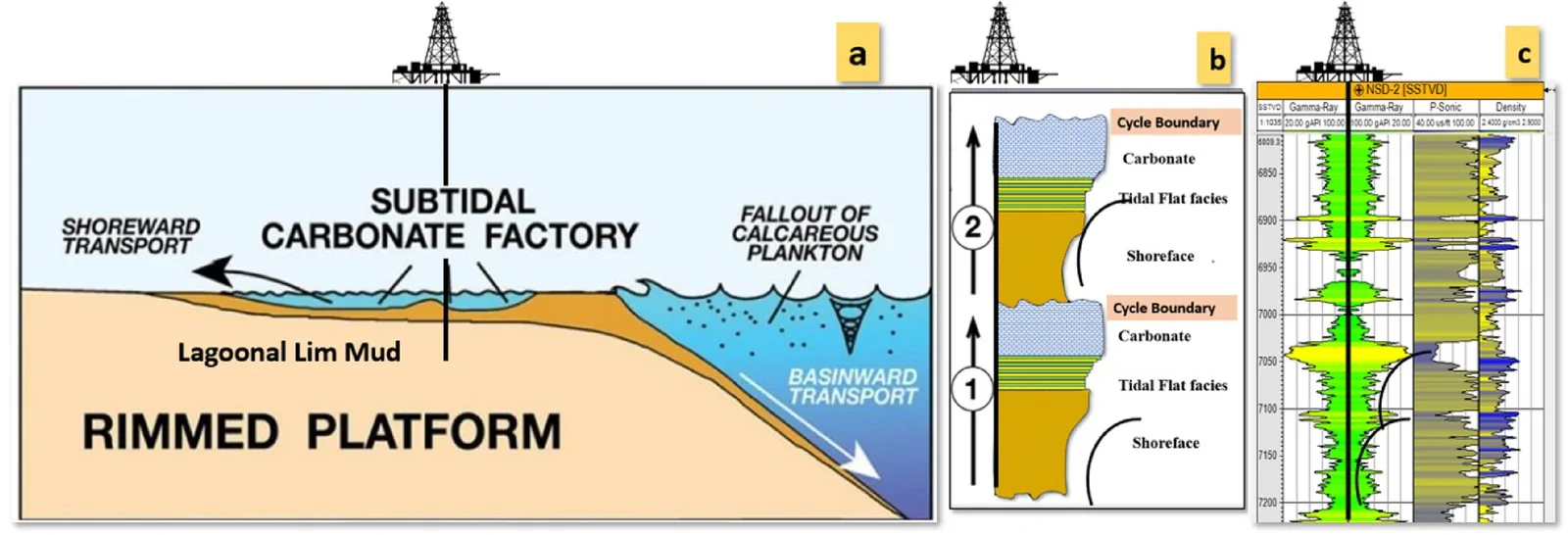
(**a**) Schematic of a rimmed carbonate platform (After^27^), analogous to carbonate deposition in the ARG Member; (**b**). cross-section illustrating lithology and cyclicity in a rimmed carbonate setting, comparable to the study area; (**c**). NSD-2 well showing density and sonic log responses corresponding to carbonate-rich intervals with the same cyclic patterns; the well log visualization was generated by the authors using Petrel.

Based on laboratory analysis, the carbonate intervals identified in the Abu Roash area (Fig.6) are composed primarily of microcrystalline lime mud with little to no fossil content. These carbonate units are consistently found interbedded between tidal flat sequences, suggesting deposition in a shallow, restricted marine environment, not within a reefal system. These facies are interpreted as part of a carbonate rimmed platform system, formed by the accumulation of sediments in an area experiencing relative subsidence. Rimmed platforms, such as the one illustrated in (Fig. 13), can span hundreds of square kilometers and reach several kilometers in thickness. These platforms may consist of stacked carbonate ramps or flat-topped geometries and are commonly associated with tidal flats, lagoons, and restricted circulation zones.

The adjacent hinterland would serve as a source of terrigenous clastic, freshwater, and nutrients. When such shallow marine systems cover vast cratons, they are termed epeiric platforms^27^. The Abu Roash “G” Member’s carbonate intervals fit within this conceptual framework of neritic, non-reefal, lime-mud dominated environments.

#### Log analysis and interpretation

**Fig. 14:**
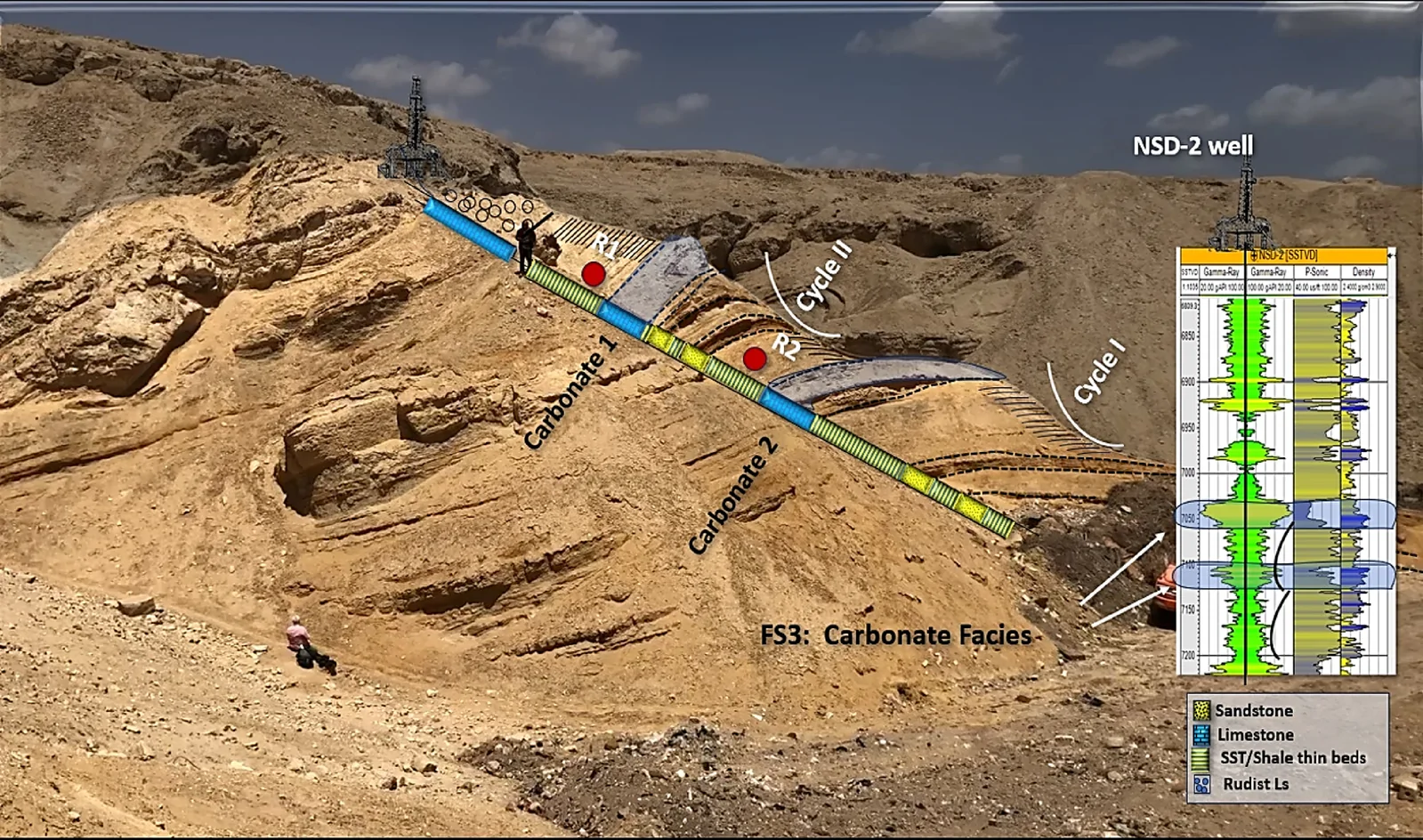
Field photograph shows the two main carbonate markers within the Abu Roash “G” Member dividing the section into two distinct cycles. Each carbonate unit caps a cycle. The tidal flat facies at the top of each cycle differ in grain size and type of sand. R1: Lime-mud tidal flat with bundled sedimentary structures; R2: Quartz-rich sandflat underlying carbonate capping.

In this case, gamma ray (GR) logs are not effective for identifying the carbonate facies due to their generally low radioactivity. Instead, sonic and density logs provide more reliable indicators. These logs confirm the presence of two distinct carbonate layers within the tidal flat sequence in the NSD-2 well (Fig.14).

The upper carbonate layer is embedded between two different types of tidal flat deposits: (a lower sandflat unit dominated by quartz grains, an upper mudflat unit characterized by carbonate-rich sediments and bundled sedimentary structures. The lower carbonate interval is also interbedded within tidal deposits, reinforcing its lagoonal origin. This stratigraphic arrangement supports the interpretation that the carbonate facies within the Abu Roash “G” Member likely represents lime mud accumulation in a restricted lagoonal setting, influenced by surrounding tidal processes. Moreover, moving northward across the study area, the thickness of these carbonate layers increases potentially extending to occupy the full vertical section of the “G” Member.

### Depositional model

#### Review of previous studies

Since the sandstone intervals in the Abu Roash “G” Member are considered prime targets for hydrocarbon production, most depositional models have been constructed with a focus on sandstone facies. Abu Roash “G” Member as primarily deposited in a near shoreface environment, Saleh et al.^21^, proposed that the Upper and Middle sections reflect a deltaic setting, while the Lower section corresponds to a tidal complex^28^, supported a deltaic model for the Middle Unit and a shallow marine environment with tidal influence for the Lower Unit. In contrast, the current study suggests that the types of tidal flat facies and carbonate sequences are the dominant controls governing the depositional model of the Abu Roash “G” Member. These facies associations provide a more refined framework for reservoir characterization.

#### Carbonate sequences

**Fig. 15:**
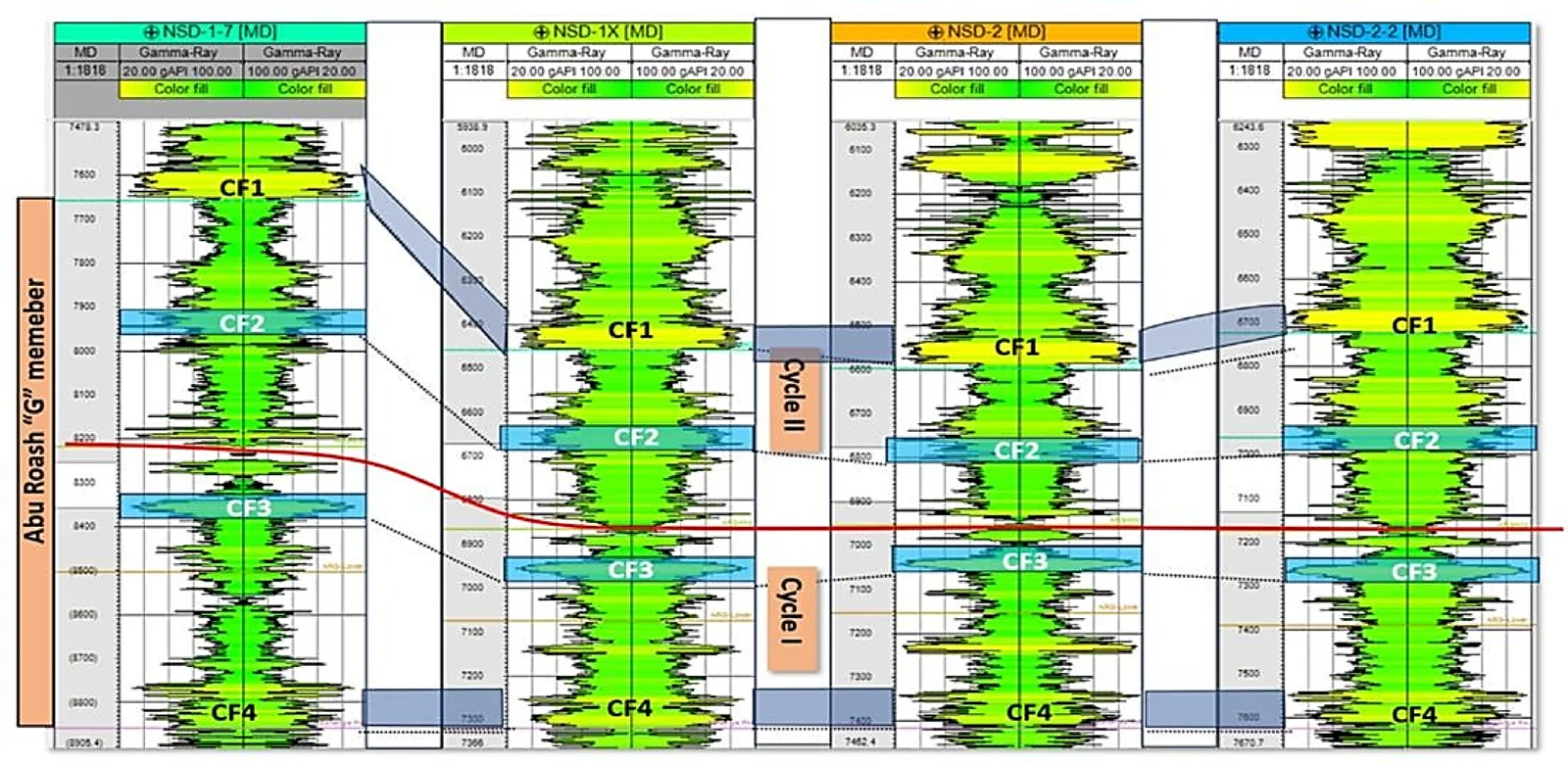
2D well correlation for the available wells, showing four carbonate markers (CF1–CF4) across the field. CF1 and CF4 occur between the Abu Roash “F” Formation (top) and the Bahariya Formation (base). CF2 and CF3 are laterally continuous markers that subdivide the Abu Roash “G” Member into two main depositional cycles, each marking the top of a cycle. Dashed lines indicate the tops of the Abu Roash “G” Member. Well log visualization was generated by the authors using Petrel.

Two-dimensional well correlation among the available wells (Fig. 15) identified four main carbonate markers (CF1, CF2, CF3, and CF4) arranged from top to bottom. These markers are laterally continuous and traceable across the field. CF4: Represents a lagoonal carbonate unit that acts as a barrier between the ARG Member and the underlying Bahariya Formation. The top of the Bahariya is interpreted as a mudflat tidal facies, supporting the idea that CF4 marks a restricted lagoonal environment as shown in (Fig. 13). CF3 and CF2: Occur at the tops of transgressive cycles and are interpreted as lagoonal carbonate caps. CF1 (Abu Roash “F”): Contains Rudists facies, indicative of a shallow marine carbonate setting.

#### Tidal flat variability (top view and cross-section)

**Fig. 16:**
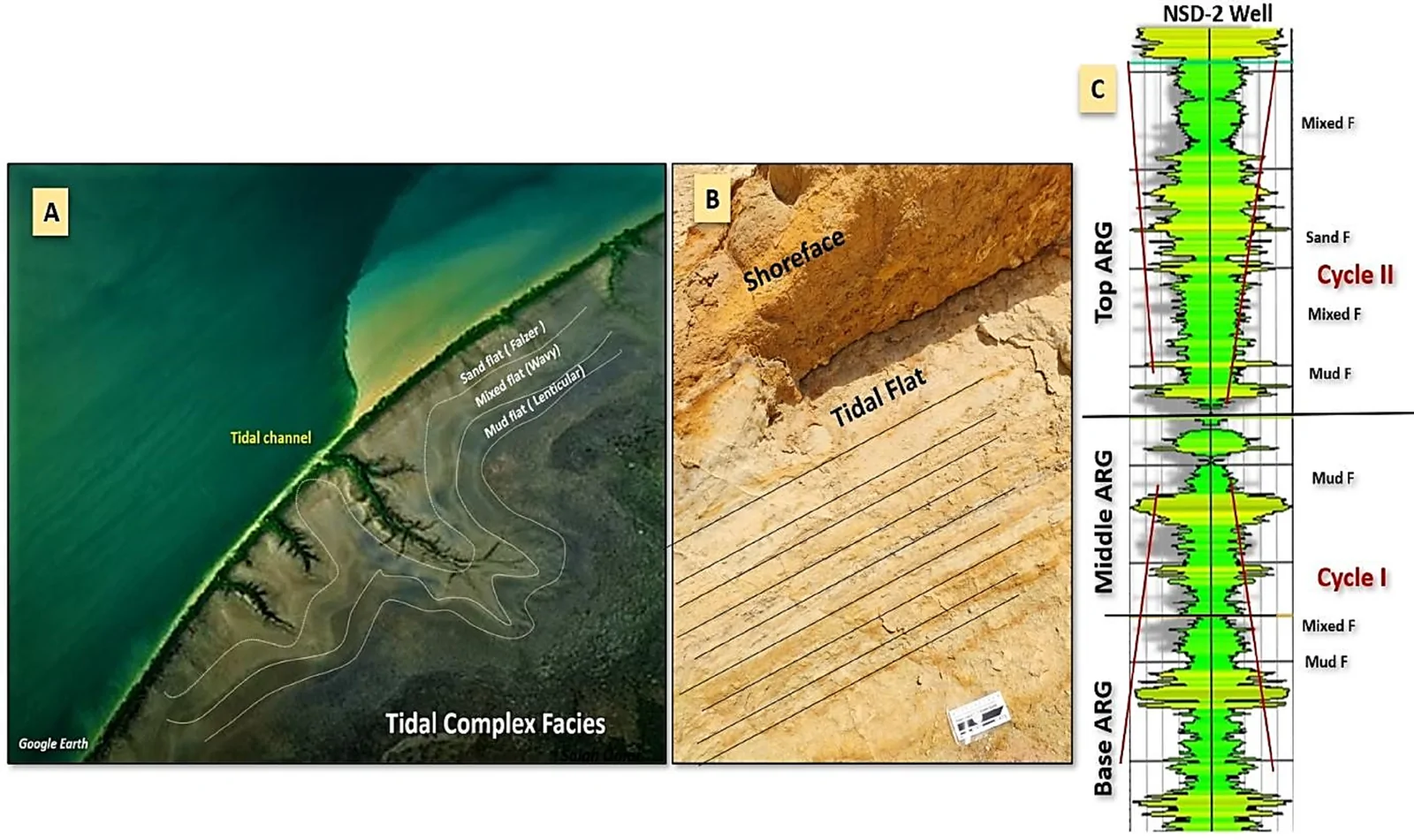
(**A**) Satellite image showing tidal channel and tidal flat facies distribution (base image from Google Earth; Imagery ss© Google), modified by the authors. (**B**) Field photograph illustrating shoreface and tidal flat deposits. (**C**) Well log from NSD-2 well showing vertical facies succession and depositional cycles within the Abu Roash “G” Member. The well log visualization was generated by the authors using Petrel.

As illustrated in (Fig.12), tidal flats can be recognized by a variety of sedimentary structures including (Mud drapes, Falser bedding, Lenticular bedding, Wavy bedding, Herringbone structures). However, bundled crossbedding is considered the most diagnostic feature for tidal flat recognition. (Fig. 16A) shows the top-view distribution of tidal flat types using satellite imagery, while (Fig. 16B) presents a cross-section from Abu Roash area, explaining their vertical and lateral relationships. A key observation is that mudflats with lenticular bedding, typically interpreted as deep marine facies based on petrophysical logs, are actually deposited landward of the shoreline when interpreted using facies concepts. Conversely, sandflats, which appear shallower in a sedimentological context, may also be misinterpreted if relying solely on log-based lithology. This reinforces the value of facies-based interpretation for understanding depositional settings over traditional lithological models.

### Abu Roash “G” Member deposional model

The Abu Roash “G” Member is considered a natural stratigraphic continuation of the underlying Bahariya Formation, forming an overall transgressive succession within a tidal complex regime.

- The shoreface facies (FS1) reflect deposition in relatively deeper marine environments, reaching up to 20 m in depth.
- The carbonate facies (FS3) are interpreted as a lagoonal carbonate, representing shallow, restricted marine conditions.
- Both facies are heavily influenced by tidal flat environments (FS2).

**Fig. 17:**
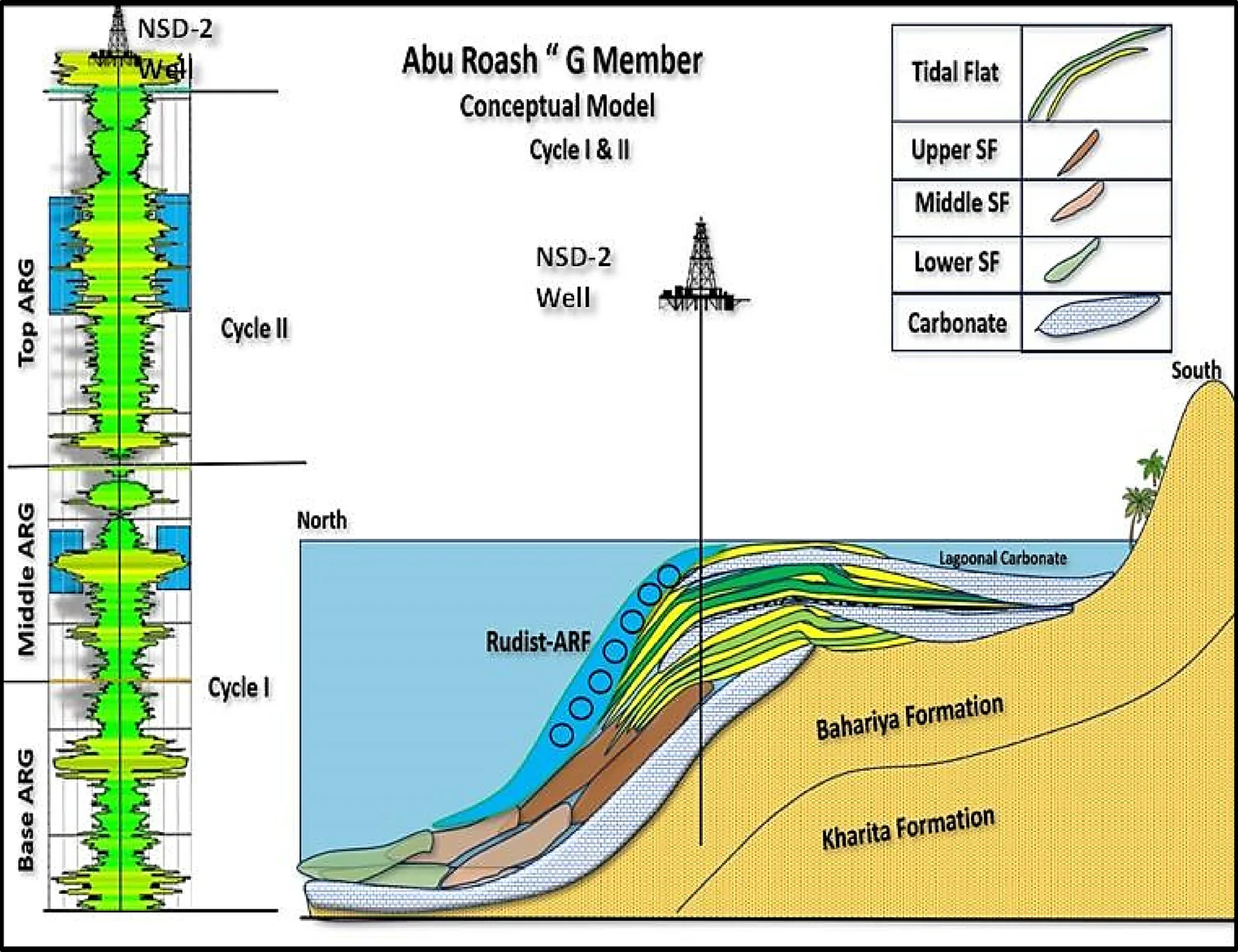
Comparison between the depositional model of the Abu Roash “G” Member and the NSD-2 well, illustrating two main transgressive cycles. The shoreface facies in Cycle I represent the primary sandstone reservoir targeted for oil production. The well log visualization was generated by the authors using Petrel.

Based on the facies associations described in earlier sections, the Abu Roash “G” Member comprises two primary transgressive cycles, with a possible unconformity separating them (Fig. 17).

#### Cycle I: main reservoir unit

In the oil industry, Cycle I include the middle and lower parts of the Abu Roash “G” Member. This cycle is characterized by the presence of yellow shoreface sandstones (FS1), which are the primary targets for oil production. Typically, only the upper shoreface facies is preserved; middle and lower shoreface deposits are less common and often influenced by tidal processes. The upper shoreface may laterally transition into middle or lower shoreface facies, which explains observed variability in well logs. While the facies can be correlated across wells, they may differ in thickness and extent due to local depositional changes or relative sea-level fluctuations.

#### Cycle II: tidal flat and carbonate dominated

**Fig. 18:**
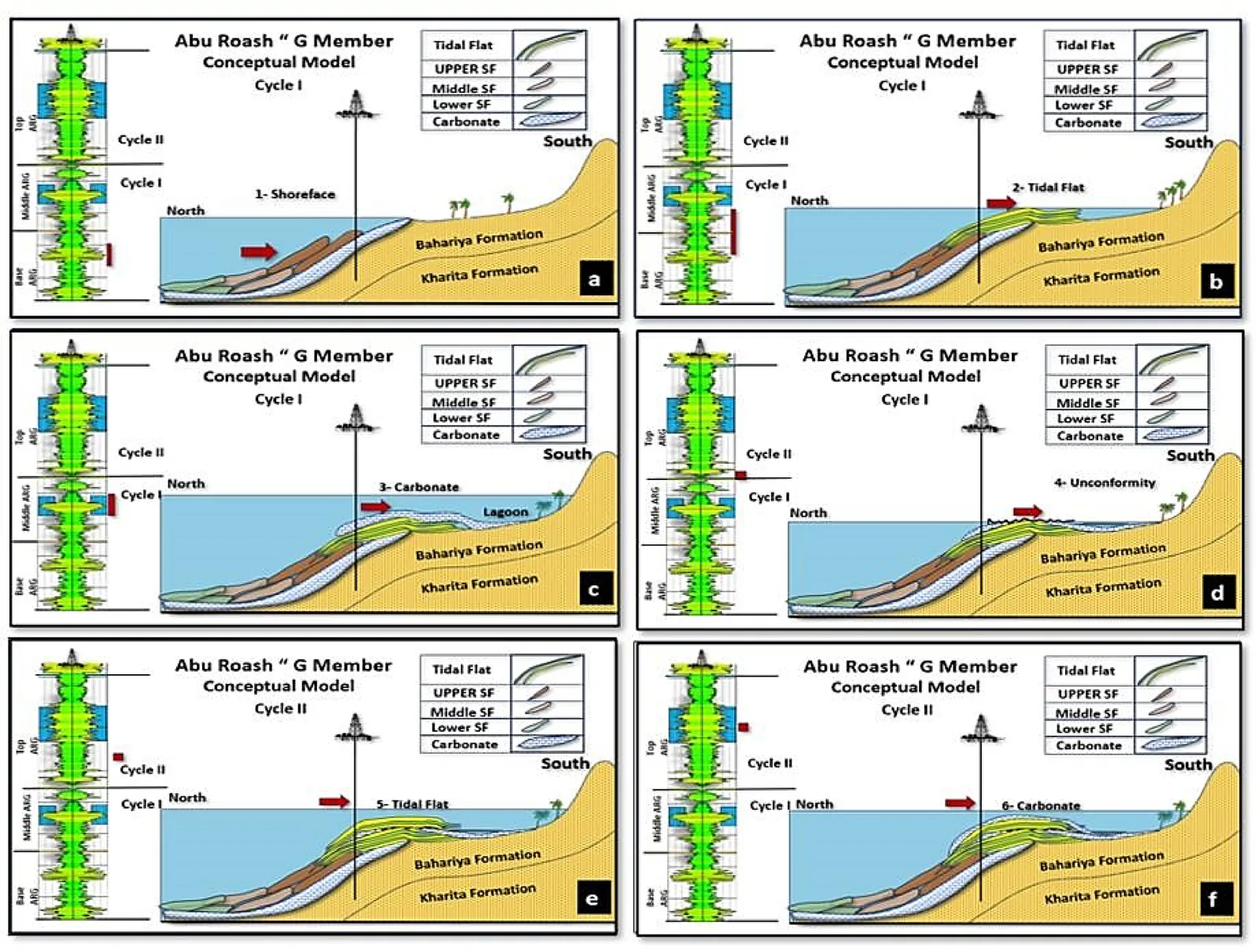
Detailed depositional model for the Abu Roash “G” Member. (**a**) Lower part of cycle I: Upper shoreface sandstone, possibly up to 20 m deep. (**b**) Continued transgression: Tidal flat deposition in shallow water. (**c**) Further transgression: Formation of a rimmed carbonate platform (restricted lagoon) with lime mud. (**d**) Relative sea-level fall, possibly due to tectonic uplift, resulting in erosion (INTR-ARG surface). (**e**) Onset of Cycle II: Deposition of sandflat tidal facies. (**f**) Transgression resumes: Restricted lagoon deposits cap the section with carbonate. The scale red bar on the well Log in each plot refers to the proposed depositional model of the indicated interval. The well log visualization was generated by the authors using Petrel.

Cycle II is dominated by tidal flat deposits, which are overlain by lagoonal carbonate layers. In some locations, thin sandstone streaks within the sandflat facies may serve as secondary reservoir targets. The detailed depositional sequence (Fig.18) aligns well with log responses from the NSD-2 well. Furthermore, it provides new insights into the stratigraphic relationships between oil company well tops (Upper, Middle, Lower Abu Roash “G”) and the two identified transgressive cycles.

### Link between surface and sub-surface well logs

Based on field analogs and the previously discussed analysis, a field-derived facies log (Fig. 20) was used as guidance for interpreting subsurface E-logs. The NSD-2 well was selected as the key reference well, as its facies interpretation closely matched the field-based predictions.

**Fig. 19:**
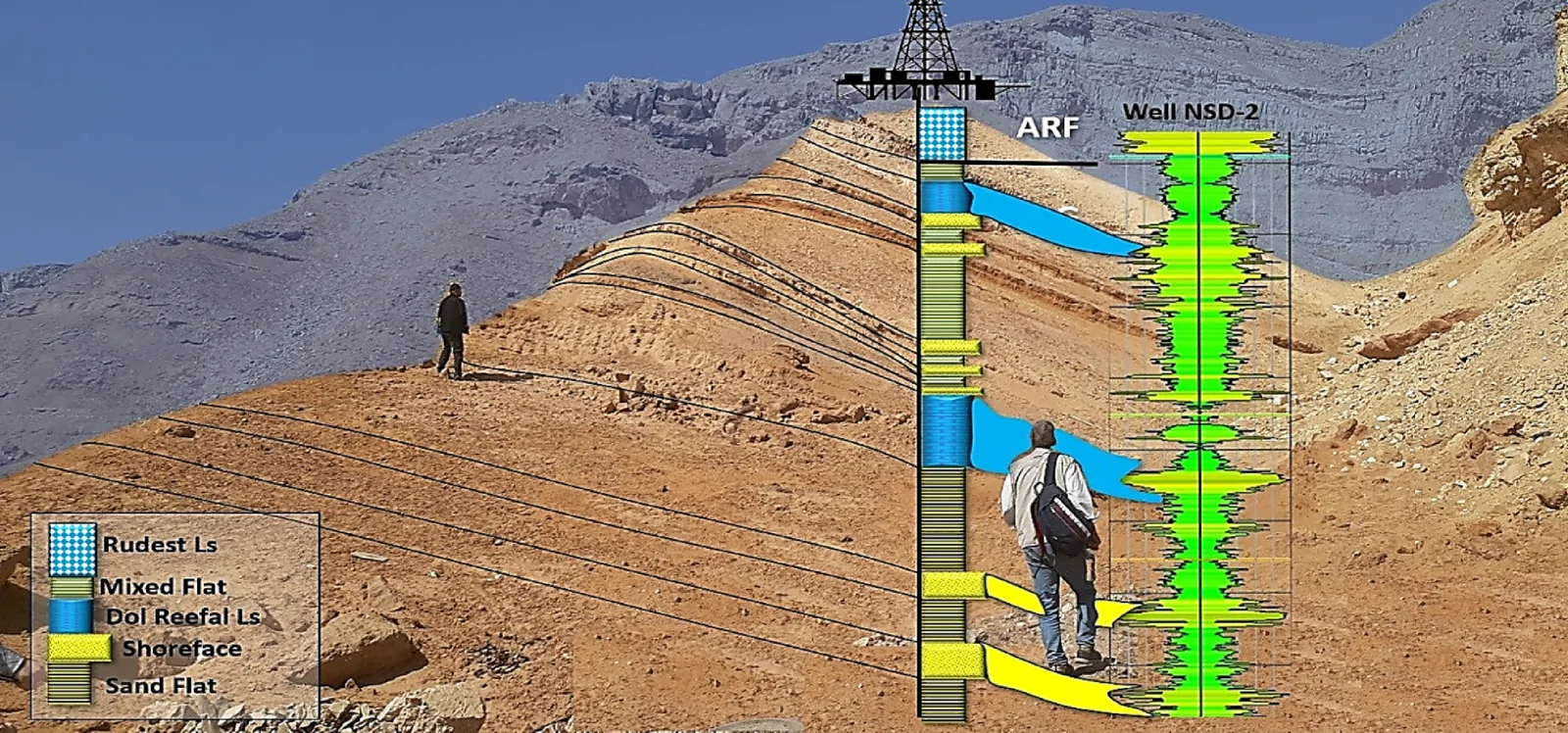
Field photograph shows a correlation between outcrop-based facies and the NSD-2 well log to be used to establish facies interpretation across other available E-logs. The well log visualization was generated by the authors using Petrel.

**Fig. 20:**
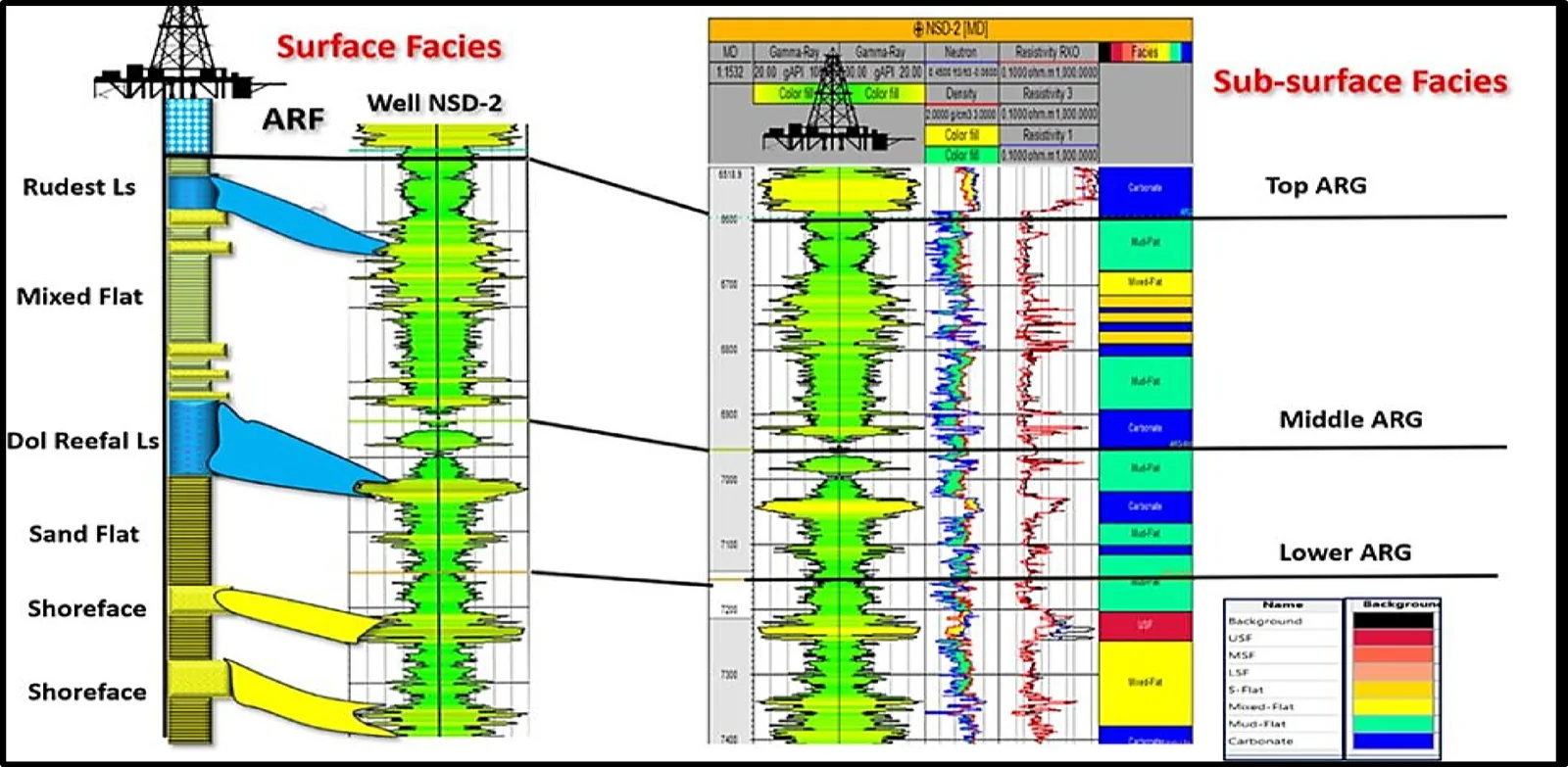
Application of field facies analogs used to construct a facies log for the NSD-2 well. The well log visualization was generated by the authors using Petrel.

**Fig. 21:**
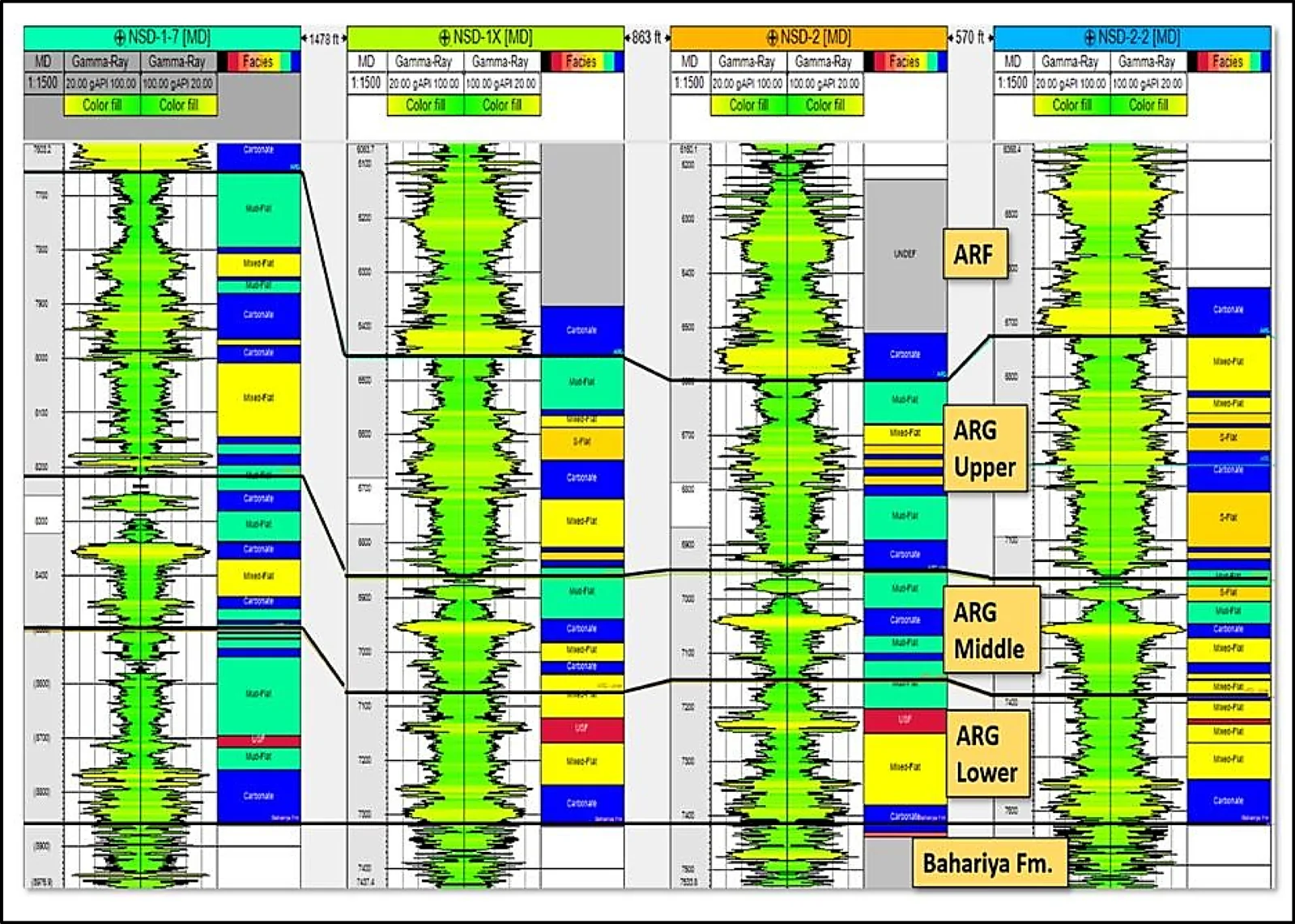
Newly generated facies log for wells NSD-2-2, NSD-1X, and NSD-1–7, using NSD-2 as a reference well. The well log visualization was generated by the authors using Petrel.

Using the NSD-2 well as a baseline, 2D well correlations were performed to extrapolate facies interpretations to three other wells: NSD-2-2, NSD-1X, and NSD-1–7 (Figs. 19–21). The previously established depositional model used as a guidance for constructing a 3D conceptual model. This facies information that derived from four wells and guided by a validated conceptual model used to build a 3D deterministic facies model for the Abu Roash “G” Member.

## Static model

A high-quality simulation model that incorporates accurate static reservoir characterization significantly improves the success rate of reservoir management and the identification of new well targets. A realistic static model plays a critical role in this process more than seismic interpretation or basic petrophysical analysis. The major challenges in building a robust static model include: (1) Gridding and layering strategies. (2) Property upscaling. (3) Facies distribution. One of the common issues is relying on lithology-based models, which can lead to random property distributions due to the lack of distinct geometry associated with lithological units. In contrast, using sedimentological facies allows for more geologically realistic modeling. The Petrel software (licensed through Alexandria University) was used for the workflow, and all outputs in this study were generated accordingly.

### Model construction approach

**Fig. 22:**
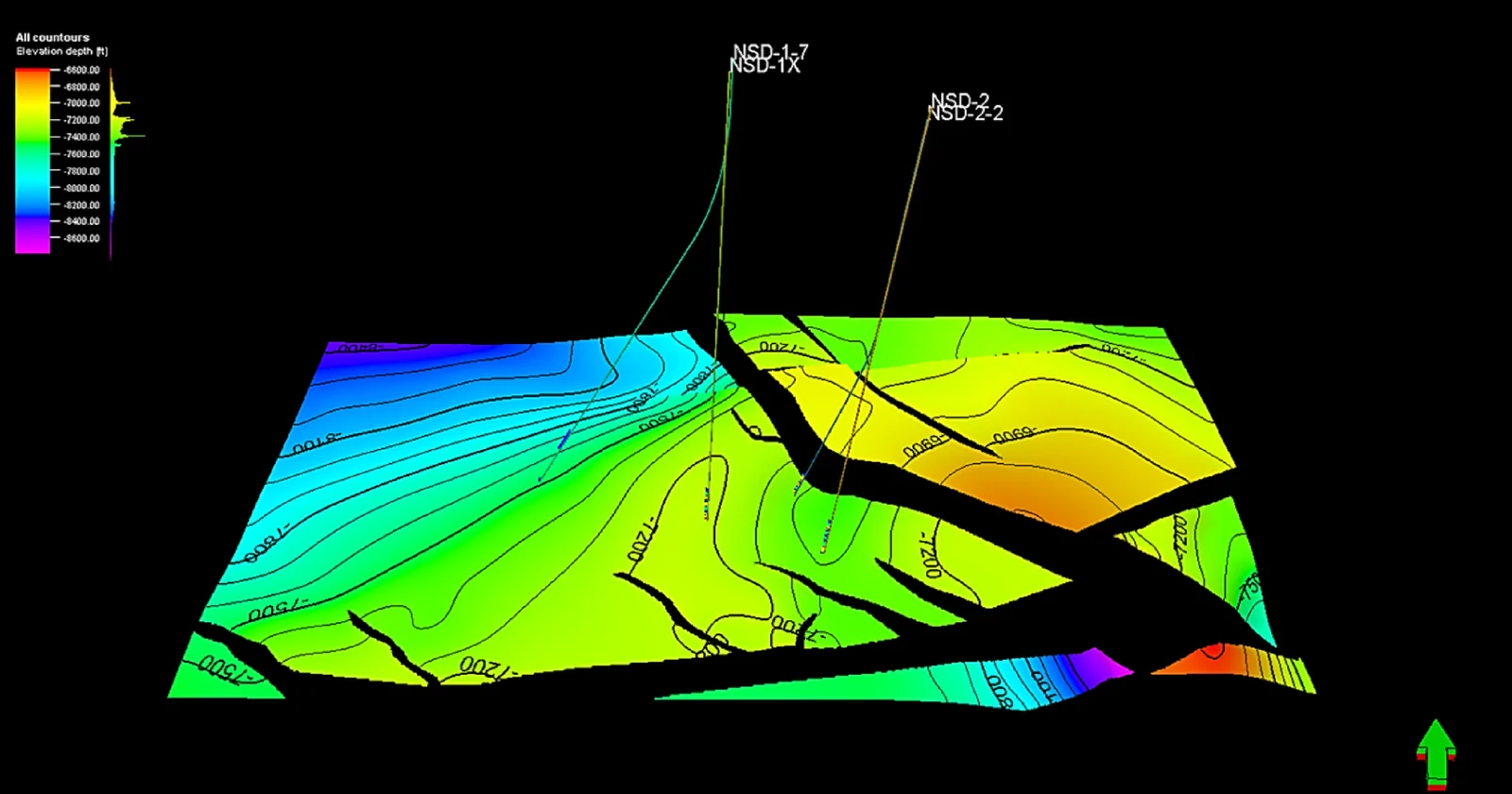
Structural depth map of the Abu Roash “G” Member, generated from seismic interpretation showing clearly delineated fault patterns that control the structural framework and influence reservoir distribution. The map was reinterpreted and generated by the authors using Petrel.

This study presents a method for deriving sedimentological facies directly from gamma ray (GR) logs by calibrating them with field outcrop analogs. The resulting facies logs were applied to develop a conceptual model, which served as the foundation for 3D facies distribution. The structural map of the Abu Roash “G” Member (Fig. 22) was generated from 2D seismic interpretation, incorporating fault patterns to define the 3D structural framework.

**Fig. 23:**
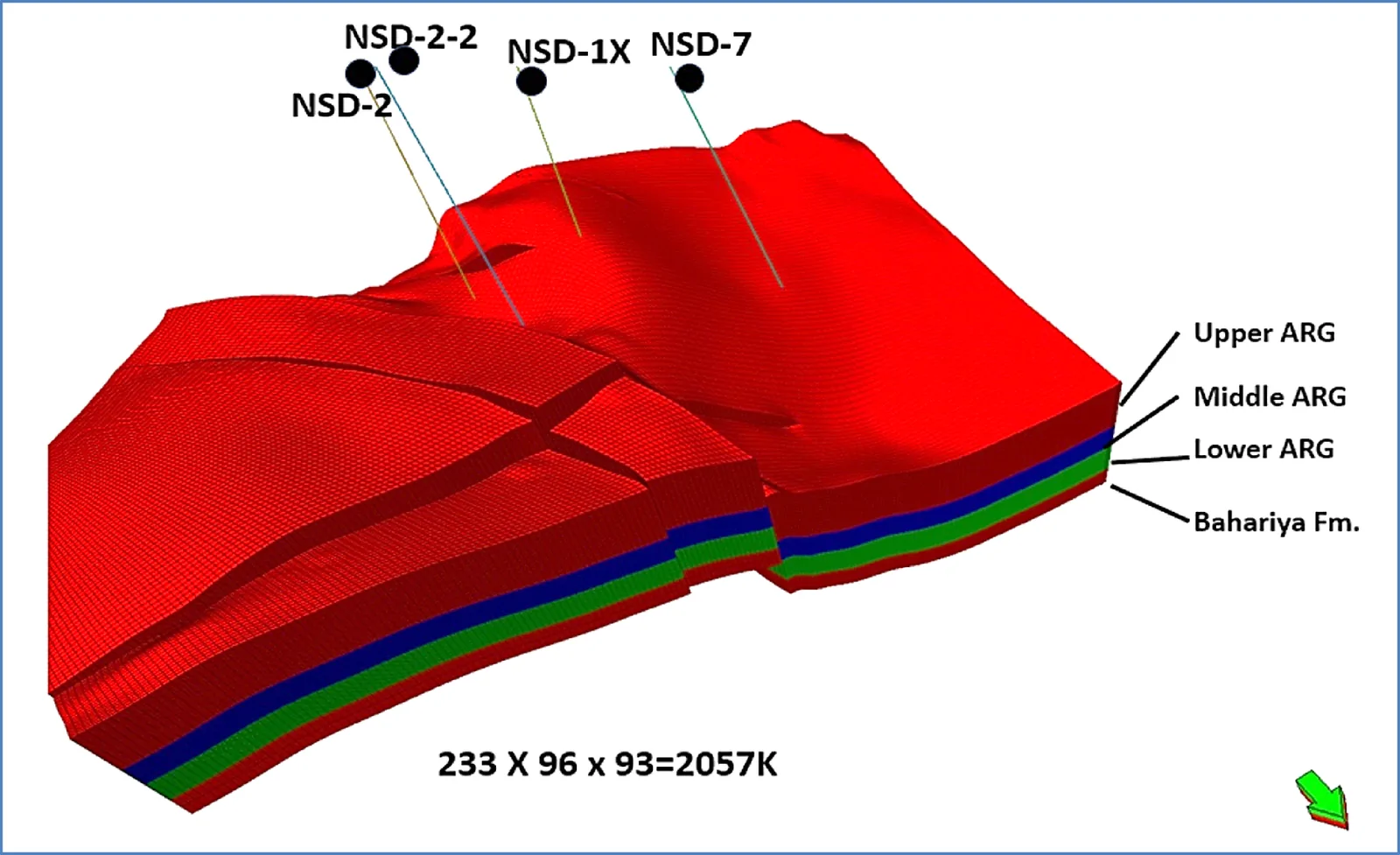
3D structural grid model consisting of approximately 2 million cells, constructed using orthogonal grids aligned with fault geometries. Facies distribution was performed manually to honor field observations and ensure geological realism. The model was generated by the authors using Petrel.

The 3D structural model (Fig. 23) was designed for use in reservoir engineering simulations, enabling control of key geological parameters. It is intended to serve as a tool for future field development planning. The model employs fine, orthogonal grids with the capacity to support dynamic simulation runs.

**Fig. 24:**
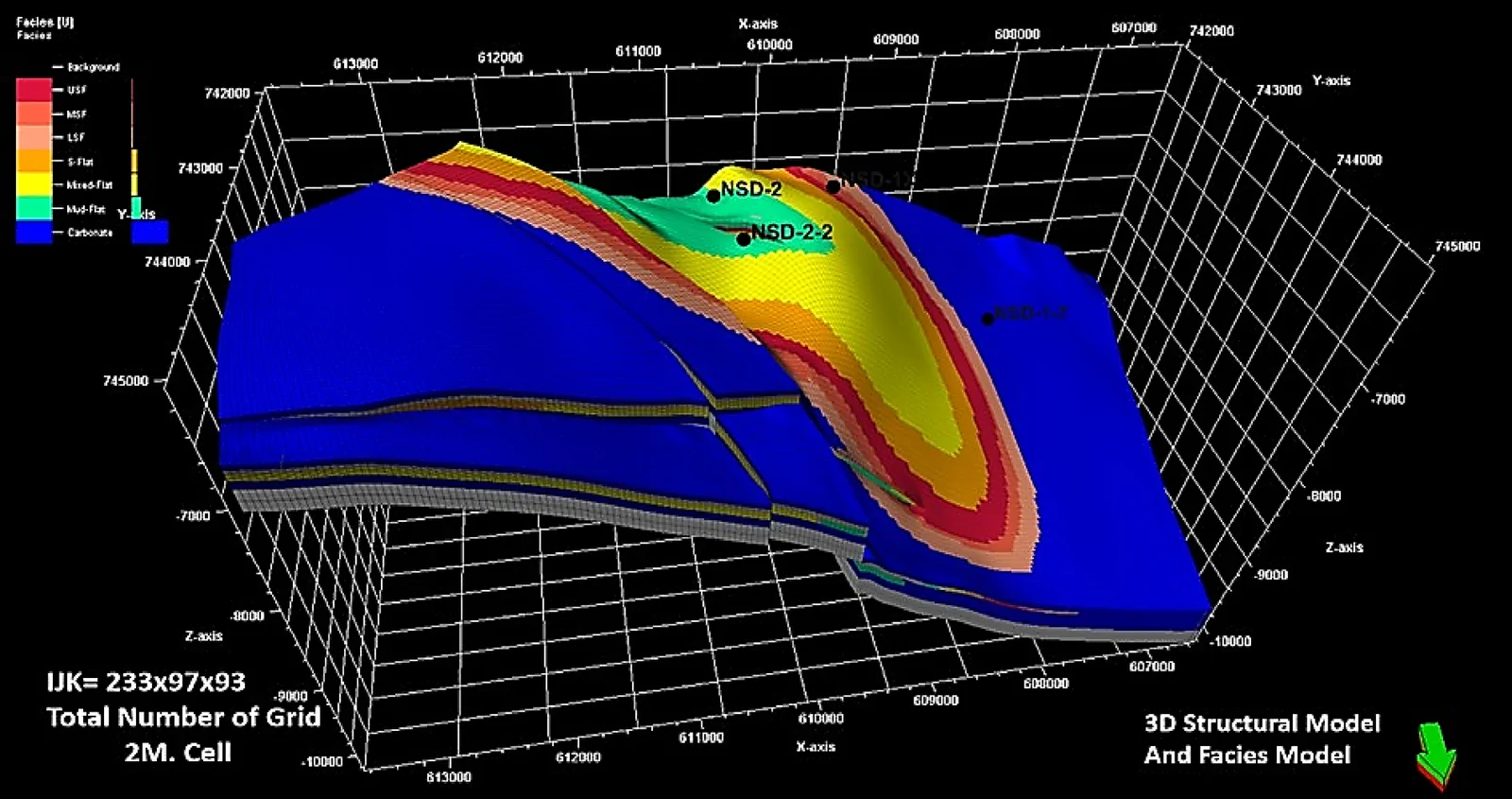
3D Facies model. The model was generated by the authors using Petrel.

**Fig. 25:**
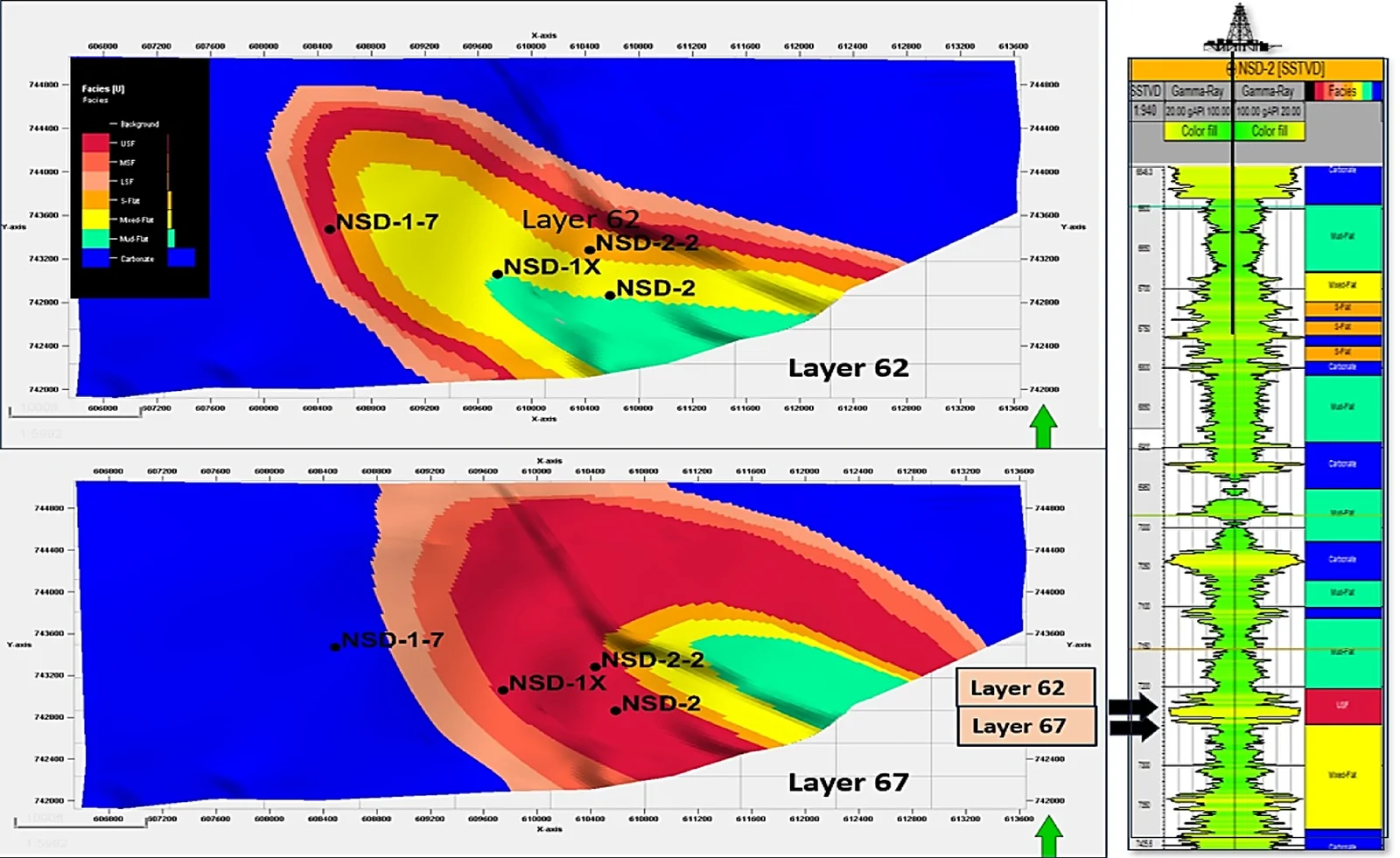
Deterministic facies distribution for representative shoreface layer (62 and 67). Facies maps and well log visualization were generated by the authors using Petrel.

**Fig. 26:**
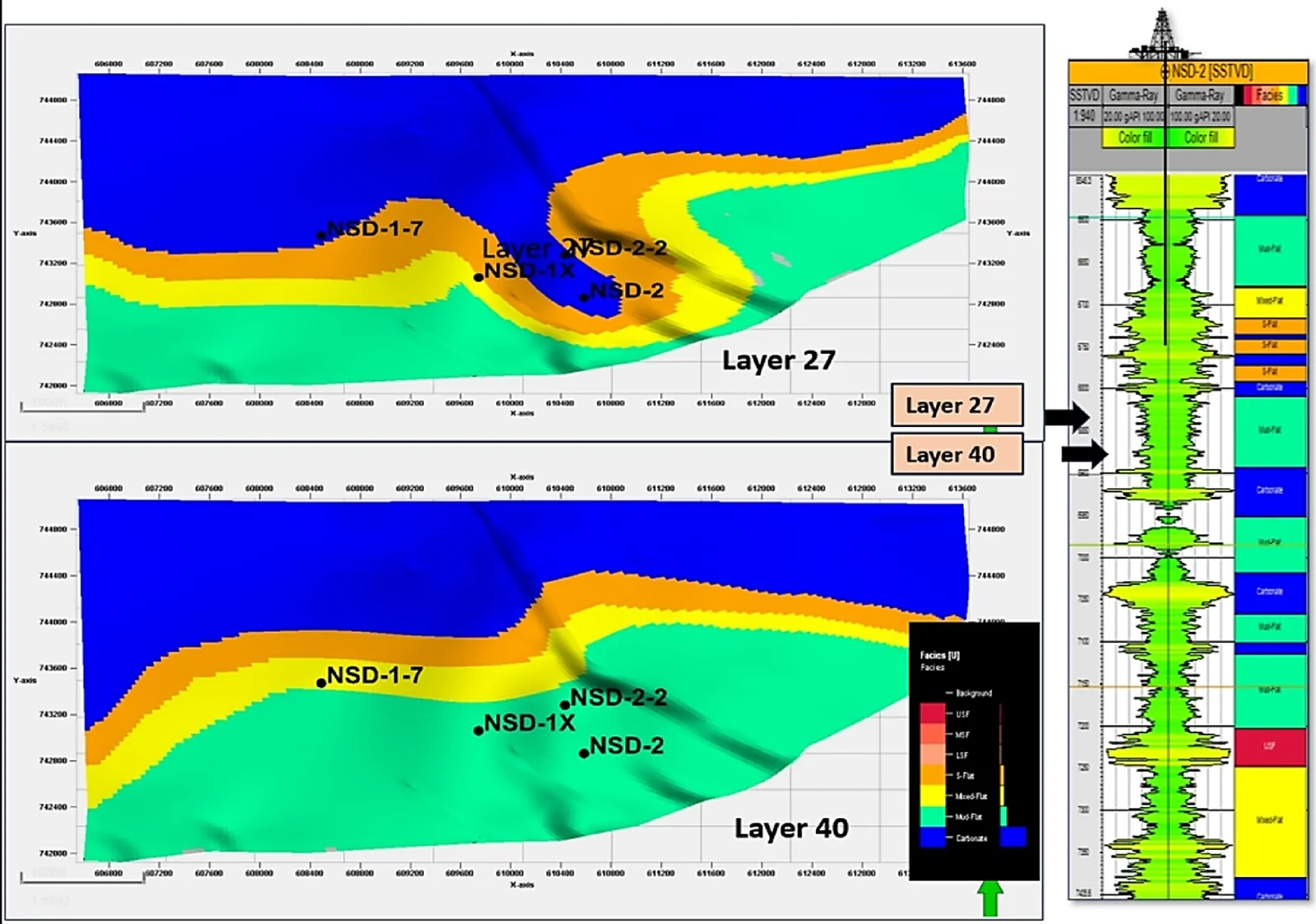
Deterministic facies distribution for representative tidal flat layers (27 and 40). Facies maps and well log visualization were generated by the authors using Petrel.

Facies were distributed using a deterministic, interactive approach (Fig. 24), manually supervised by the interpreter across nearly 99 model layers to capture detailed vertical facies variability. Facies distribution was constrained to well-controlled areas and guided by the conceptual depositional model shown in Fig. 17. (Figures 25 and 26) present examples of detailed facies distribution for a shoreface layer and selected tidal-flat layers, respectively, demonstrating good agreement with the facies interpretation of the key well NSD-2.

**Fig. 27:**
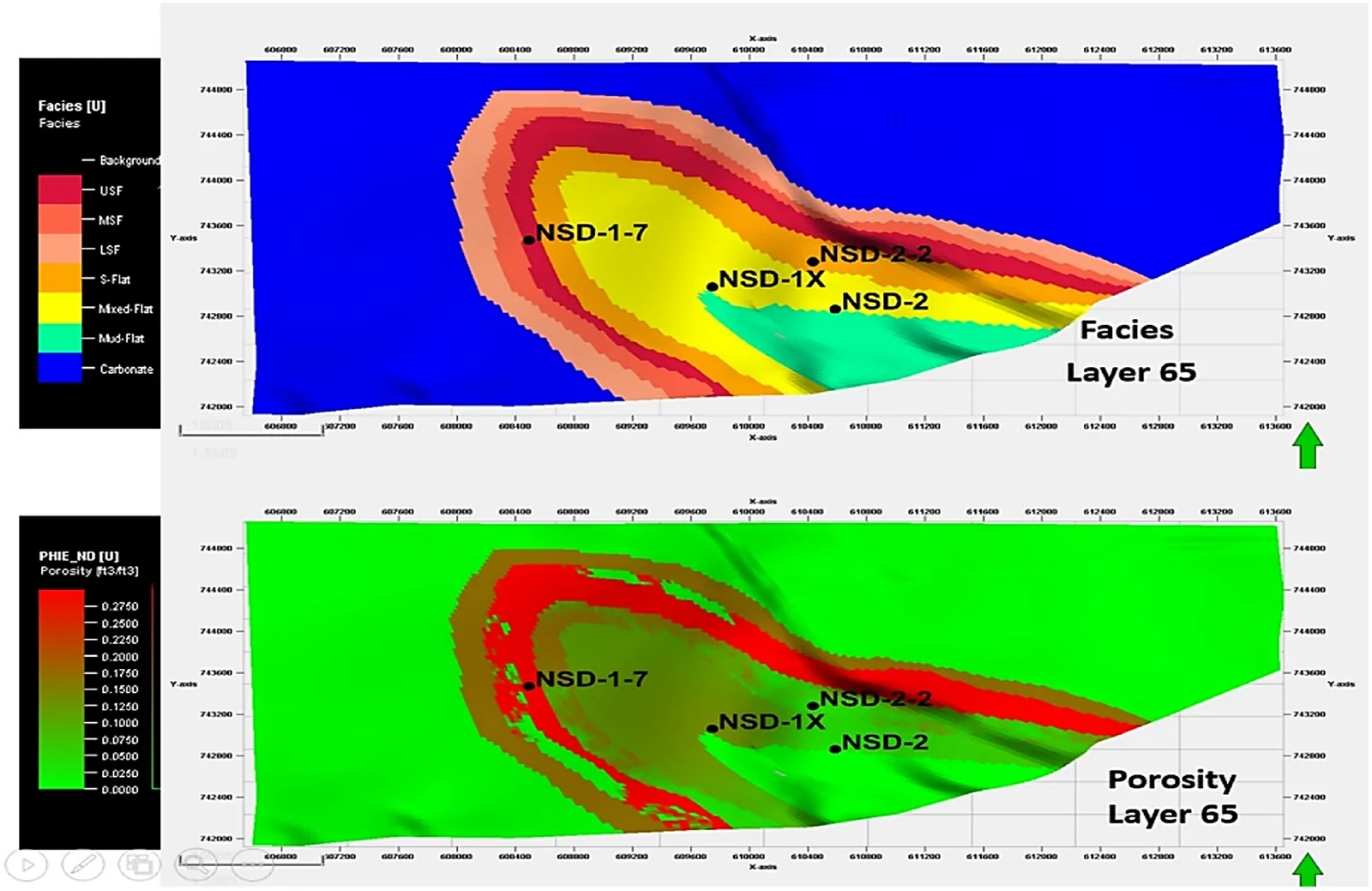
Comparison between the facies distribution and porosity model in Layer 65, shown as a representative example. The facies and porosity models were generated by the authors using Petrel.

The three-dimensional porosity model was generated as part of the static reservoir modeling workflow using interpreted porosity logs derived from petrophysical analysis. Porosity population and spatial distribution were constrained by the three-dimensional depositional facies model to ensure consistency between sedimentological architecture and reservoir properties. Shoreface facies, characterized by high effective porosity and favorable textural attributes, represent the primary reservoir units within the model. Sand-flat facies exhibit moderate porosity values and form secondary reservoir intervals with localized storage potential. (Fig. 27) illustrates the linkage between facies logs along a representative two-dimensional cross section and the resulting three-dimensional porosity distribution, demonstrating the strong facies control on static property modeling.

**Fig. 28:**
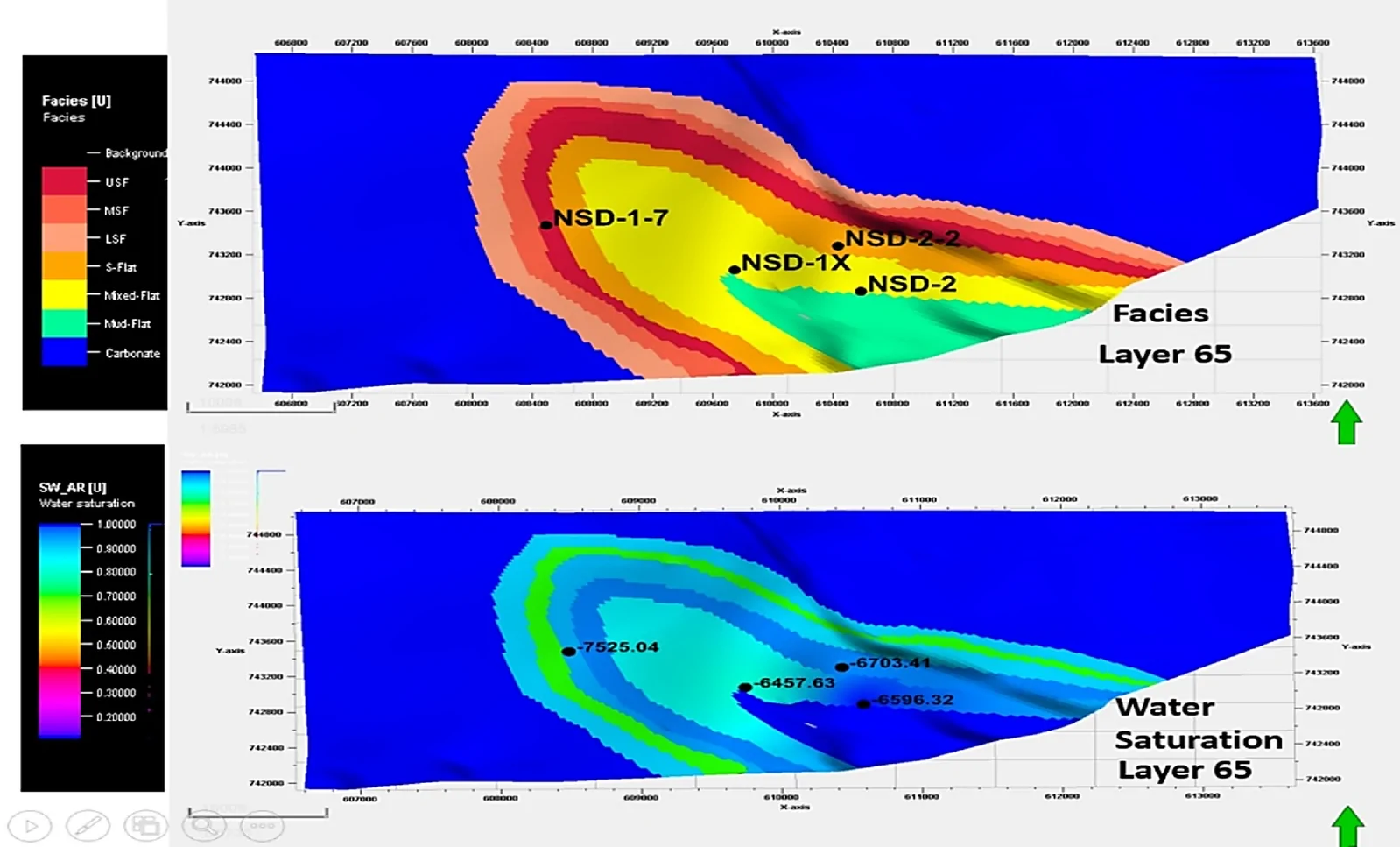
Comparison between the facies distribution and Water saturation model in Layer 65, shown as a representative example. The facies and porosity models were generated by the authors using Petrel.

The water saturation model (Fig. 28) within the static reservoir framework was generated using the available log-derived water saturation data from the studied wells. However, the absence of a clearly defined water–oil contact (WOC), together with the fact that the existing wells do not represent initial reservoir conditions, introduces significant uncertainty in the estimation of true in-situ water saturation. Consequently, the modeled water saturation distribution should be regarded as a relative representation rather than an exact depiction of original reservoir saturation conditions. Future incorporation of additional water saturation constraints and key reservoir engineering parameters (e.g., capillary pressure data, relative permeability functions, and dynamic pressure information) is expected to improve volumetric estimates and enhance the readiness of the static model for subsequent dynamic simulation.

**Fig. 29:**
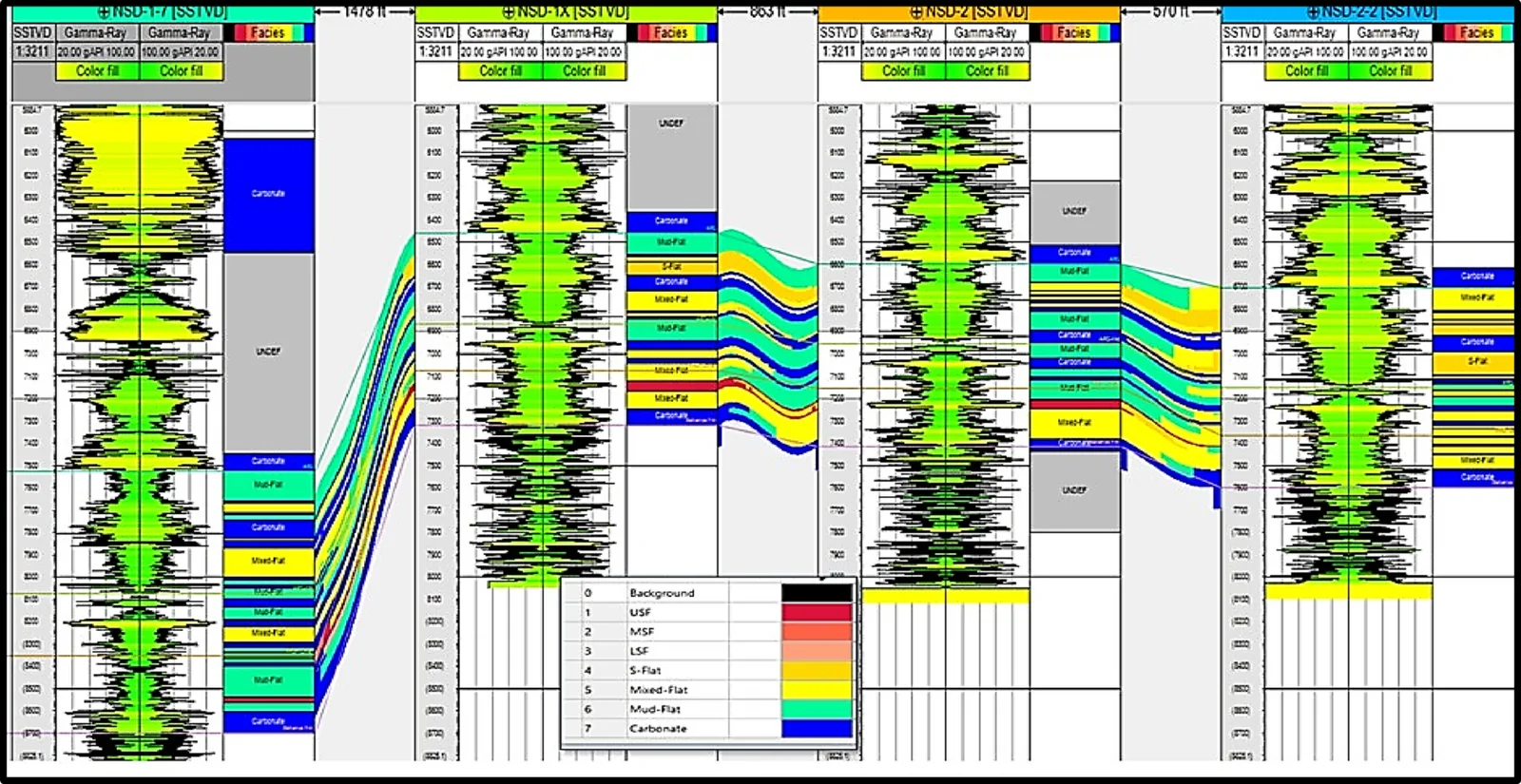
The 2D well correlation illustrating the final 3D facies distribution, showing a close match between well log facies and the background lateral facies patterns. The 3D facies model and log visualization were generated by the authors using Petrel.

**Fig. 30:**
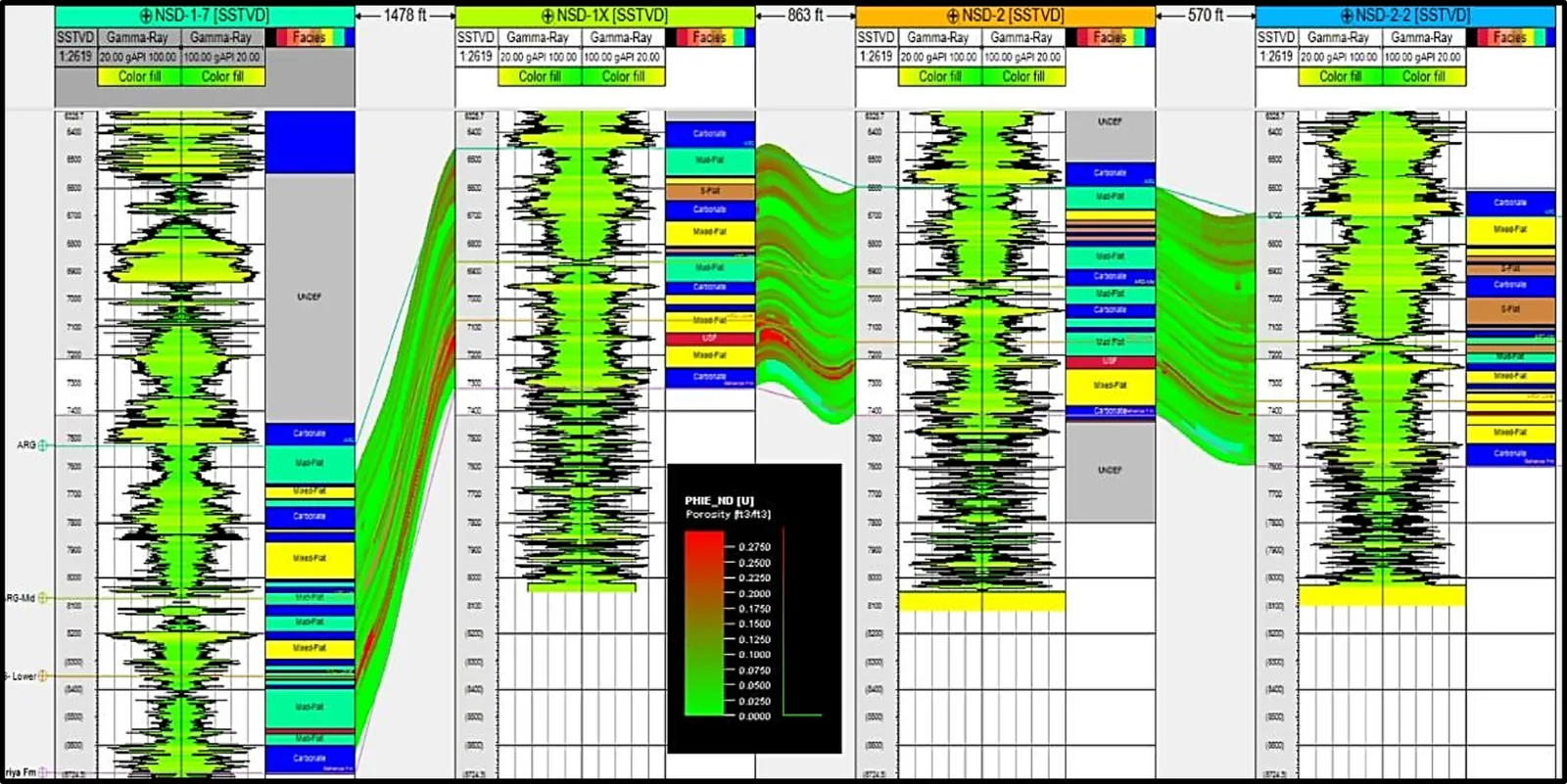
Two-dimensional well correlations of porosity logs with the three-dimensional porosity distribution shown in the background. The 3D facies model and log visualization were generated by the authors using Petrel.

Two-dimensional well correlations of facies (Fig. 29) and porosity logs (Fig. 30) were constructed based on the newly interpreted facies logs, with the corresponding three-dimensional facies and porosity distributions displayed in the background. The background representation of the three-dimensional facies model highlights spatial facies consistency and supports the interpretation of depositional trends across the study area. Additionally, the integration of two-dimensional porosity log correlations with the three-dimensional porosity distribution suggests a relatively consistent lateral porosity trend, largely governed by the deterministic facies’ distribution.

**Fig. 31:**
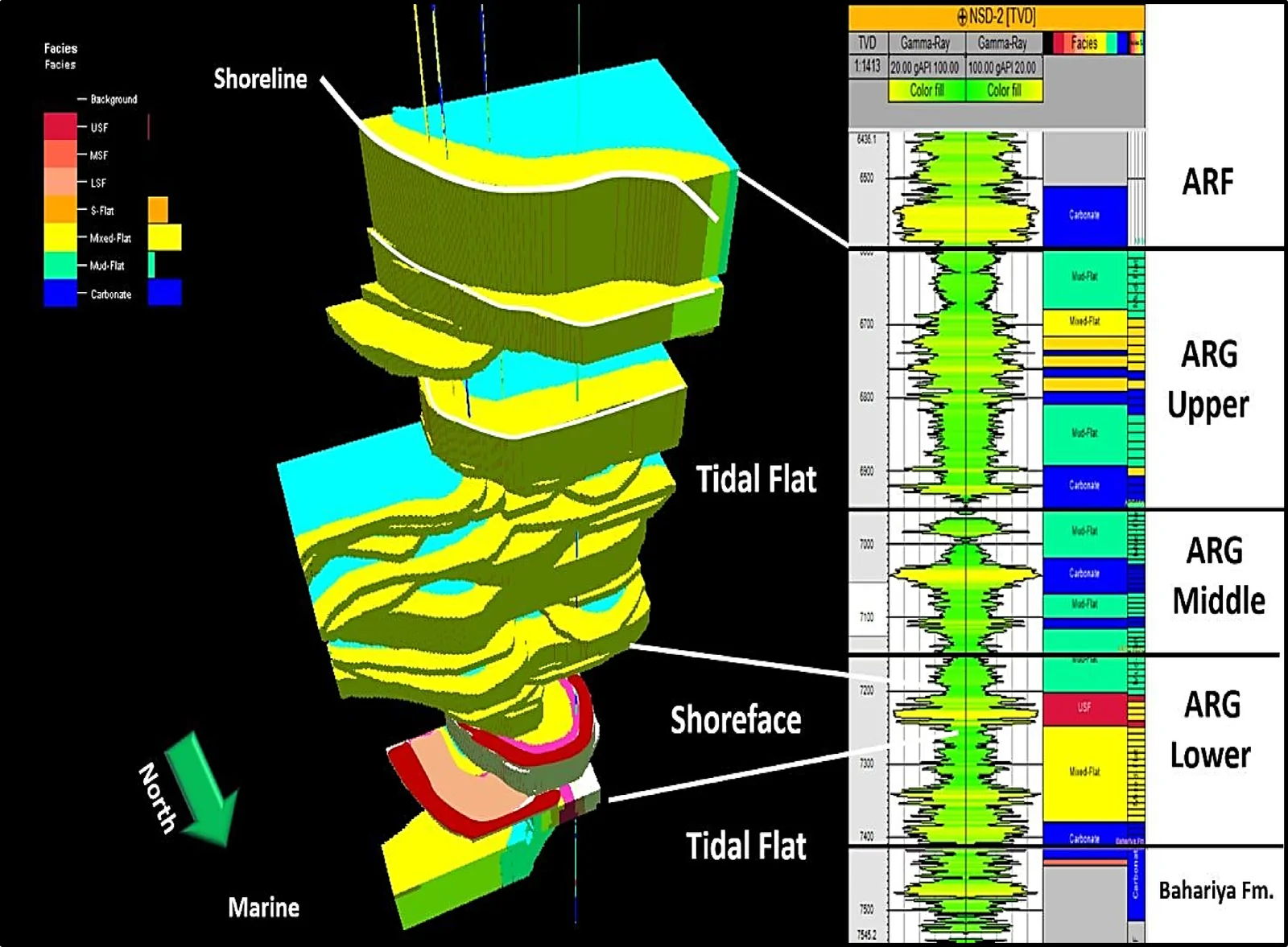
2D well correlation based on the newly interpreted facies logs. The background displays the resulting 3D porosity model, which supports the validation of reservoir continuity and quality across the correlated wells. The 3D facies model and log visualization were generated by the authors using Petrel.

One of the key advantages of distributing facies using a deterministic approach is the ability to document and analyze shoreline movement over time. The 3D image (Fig. 31) illustrates fluctuating tidal flat facies at the base of the Lower Abu Roash “G” Member, indicating a shallow marine environment with strong tidal influence. A lower transgressive cycle follows, increasing water depth and leading to the deposition of shoreface sediments. Slight tectonic activity may have contributed to changes in shoreline geometry. The Middle and Upper ARG members continue to show sea-level fluctuations under tidal influence. Log curves and facies distributions correlate well with this shoreline movement.

## Conclusion

High-resolution reservoir modeling in heterolithic siliciclastic–carbonate successions require explicit integration of sedimentological facies architecture with petrophysical property distribution. Log-based electrofacies classification alone is commonly non-unique in tidally influenced systems, owing to overlapping gamma-ray responses among sand-rich and mud-rich facies, which can lead to oversimplified reservoir geometries. To address this limitation, the present study applies a facies-conditioned gamma-ray simulation workflow in which outcrop-derived facies associations are quantitatively linked to characteristic gamma-ray motifs and subsequently used for subsurface calibration.

The Abu Roash area, as the type locality of the Abu Roash “G” (ARG) Member, provides an exceptional analog for integrating surface and subsurface datasets. Detailed outcrop analysis indicates that the ARG Member is organized into three stratigraphic intervals—lower, middle, and upper—bounded by laterally continuous carbonate units that record major shifts in accommodation. These carbonate horizons are interpreted as representing lagoonal environments. Facies stacking patterns reflect the interaction of two coeval depositional systems: a wave-dominated shoreface system responsible for the accumulation of laterally extensive, well-sorted sandstone bodies, and a tidally influenced flat system that imposed heterolithic textures and vertical facies alternations throughout the succession.

Five facies’ associations were identified in the outcrop section: FS1 (upper–lower shoreface sandstone), FS2 (sand flat), FS3 (mixed flat), FS4 (mud flat), and FS5 (lagoonal carbonate). Each facies association was assigned a characteristic gamma-ray response envelope based on grain size, clay content, and biogenic contribution. These envelopes were integrated to generate a synthetic gamma-ray log for the outcrop succession, which was subsequently used as a calibration template for facies interpretation in the NSD-2 well. The calibrated facies log was then propagated to the NSD-1, NSD-2-2, and NSD-1–7 wells through chronostratigraphic correlation constrained by carbonate marker beds.

Outcrop-derived facies proportions, vertical transition probabilities, and lateral facies relationships were incorporated into a deterministic static reservoir model. Facies distribution rules were constrained by shoreline orientation, paleogeographic trends, and satellite imagery (e.g., Google Earth), allowing for realistic reconstruction of shore-parallel sandstone bodies and landward-directed tidal-flat facies belts. This approach ensures spatial continuity consistent with depositional processes rather than purely stochastic interpolation.

The results demonstrate that the conventional industry subdivision of the Abu Roash “G” Member into Top, Middle, and Lower units—commonly based on sandstone occurrence—does not adequately capture the genetic stratigraphic framework. Instead, the proposed facies-based model provides improved prediction of reservoir connectivity, facies continuity, and vertical flow barriers. Furthermore, the integration of facies-conditioned gamma-ray modeling enables tracking of shoreline migration and accommodation shifts through time, offering critical insights into the combined influence of relative sea-level change and tectonic activity on reservoir development in the Abu Roash area.

## Data Availability

The data that supports the findings of this study are available from the Egyptian General Petroleum Corporation and Petrosilah Petroleum Company, but restrictions apply to the availability of these data.
